# Clinical features, MRI, molecular alternations, and prognosis of astrocytoma based on WHO 2021 classification of central nervous system tumors: A single‐center retrospective study

**DOI:** 10.1002/cam4.7369

**Published:** 2024-07-05

**Authors:** Yuekun Wang, Hao Xing, Xiaopeng Guo, Wenlin Chen, Yaning Wang, Tingyu Liang, Hai Wang, Yilin Li, Shanmu Jin, Yixin Shi, Delin Liu, Tianrui Yang, Yu Xia, Junlin Li, Jiaming Wu, Qianshu Liu, Tian Qu, Siying Guo, Huanzhang Li, Kun Zhang, Yu Wang, Wenbin Ma

**Affiliations:** ^1^ Department of Neurosurgery, Center for Malignant Brain Tumors, National Glioma MDT Alliance Peking Union Medical College Hospital, Chinese Academy of Medical Sciences and Peking Union Medical College Beijing China; ^2^ China Anti‐Cancer Association Specialty Committee of Glioma Beijing China; ^3^ '4+4' Medical Doctor Program Chinese Academy of Medical Sciences and Peking Union Medical College Beijing China; ^4^ Eight‐year Medical Doctor Program Chinese Academy of Medical Sciences and Peking Union Medical College Beijing China; ^5^ Tsinghua University Ringgold standard institution School of Medicine, Tsinghua University Beijing China

**Keywords:** astrocytoma, FGFR2, integrate diagnoses, WHO classification, WHO grade 4 astrocytoma

## Abstract

**Background:**

The diagnosis of glioma has advanced since the release of the WHO 2021 classification with more molecular alterations involved in the integrated diagnostic pathways. Our study aimed to present our experience with the clinical features and management of astrocytoma, IDH mutant based on the latest WHO classification.

**Methods:**

Patients diagnosed with astrocytoma, IDH‐mutant based on the WHO 5th edition classification of CNS tumors at our center from January 2009 to January 2022 were included. Patients were divided into WHO 2–3 grade group and WHO 4 grade group. Integrate diagnoses were retrospectively confirmed according to WHO 2016 and 2021 classification. Clinical and MRI characteristics were reviewed, and survival analysis was performed.

**Results:**

A total of 60 patients were enrolled. 21.67% (13/60) of all patients changed tumor grade from WHO 4th edition classification to WHO 5th edition. Of these, 21.43% (6/28) of grade II astrocytoma and 58.33% (7/12) of grade III astrocytoma according to WHO 4th edition classification changed to grade 4 according to WHO 5th edition classification. Sex (*p* = 0.042), recurrent glioma (*p* = 0.006), and Ki‐67 index (*p* < 0.001) of pathological examination were statistically different in the WHO grade 2–3 group (*n* = 27) and WHO grade 4 group (*n* = 33). CDK6 (*p* = 0.004), FGFR2 (*p* = 0.003), and MYC (*p* = 0.004) alterations showed an enrichment in the WHO grade 4 group. Patients with higher grade showed shorter mOS (mOS = 75.9 m, 53.6 m, 26.4 m for grade 2, 3, and 4, respectively, *p* = 0.01).

**Conclusions:**

Patients diagnosed as WHO grade 4 according to the 5th edition WHO classification based on molecular alterations are more likely to have poorer prognosis. Therefore, treatment should be tailored to their individual needs. Further research is needed for the management of IDH‐mutant astrocytoma is needed in the future.

## 1 INTRODUCTION

Glioma is the most common primary brain tumors in adults. The classification of brain tumor has changed rapidly in the last 2 decades, since genetic variants were added in the 3rd edition of the World Health Organization (WHO) classification of central nervous system (CNS) tumors in 2000.^1^ In the 2016 WHO classification of CNS tumors, molecular features were first integrated into diagnoses. Diffuse astrocytic tumors were acknowledged as diffuse astrocytoma (grade II), anaplastic astrocytoma (grade III), and glioblastoma (grade IV) with isocitrate dehydrogenase (IDH)‐mutant, IDH wildtype and not otherwise specified (NOS) and diffuse midline glioma with H3K27M‐mutant.^2^

The classifications of astrocytic glioma were mainly determined by pathology and IDH gene variants. CDKN2A homozygous deletion, PDGFRA amplification, and CDK4 amplification were found to be associated with shorter progression‐free survival (PFS) and overall survival (OS) in WHO grade II–III astrocytoma with IDH‐mutant.^3^ Glioblastoma with IDH‐wildtype also had a worse prognosis than IDH‐mutant.^4^ In the new edition of the classification of CNS tumors,^5^ astrocytic tumors were diagnosed as astrocytoma, IDH‐mutant and glioblastoma, IDH‐wildtype. Astrocytoma with IDH‐mutant and microvascular proliferation and/or necrosis and/or CDKN2A/B homozygous deletion was classified as WHO grade 4.

Modified treatment and prognosis should be clarified under the new classification. In our previous report, we reported experiences with histological and molecular WHO Grade 4 astrocytoma according to the WHO 5th classification. Our findings indicated that histological WHO Grade 4 astrocytoma is predisposed to show an enhanced lesion on contrast‐enhanced MRI, while more than half of cases of molecular WHO Grade 4 astrocytoma exhibit a non‐enhanced pattern. We also found that alterations in PIK3R1, Notch1, and Mycn are associated with the overall survival of molecular WHO Grade 4 astrocytoma.^6^ In practice, under the new WHO 2021 classification, the molecular markers input in diagnosis often mandates a quick change in patients' treatment. Patients initially diagnosed with glioblastoma (per WHO CNS4) that switched to astrocytoma, IDH‐mutant, grade 4 (per WHO CNS5) experienced disease much easier to control even after relapse, with full‐dose reirradiation and chemotherapy.^7^ Also, for those changed from grade II–III astrocytoma (CNS4) to grade 4 astrocytoma (CNS5), enhanced treatment needed further clinical practices and researches. In this study, we respectively reviewed a group of patients with astrocytoma with 2021 classifications, and summarized the clinical and molecular features and prognoses.

## 2 MATERIALS AND METHODS

### 2.1 Patients and data collection

The study included patients who underwent craniotomy or biopsy at our center and were diagnosed with IDH‐mutant astrocytoma of WHO grade 2–4, based on the integrated diagnosis according to the WHO 2021 classification of CNS tumors between January 2009 and January 2022 were included. A consecutive cohort was established, and patients were interviewed at 1, 3, 6, 9, and 12 months after surgery, and every 6 months thereafter until tumor recurrence. Patient characteristics were collected from the medical record system and follow‐up, including general information, clinical manifestations, MRI features, pathological diagnosis, treatments, and survival interview. All patients underwent a 1.5 or 3.0 Tesla contrast‐enhanced brain MRI at 1 day to 1 week before neurosurgery or biopsy. T1‐weight, T2‐weight, and contrast T1 sequence were done for each of patients. The MRI features were determined through discussion between a neuroradiologist and neurosurgeon. Our study received approval from the Institutional Review Board of Peking Union Medical College Hospital, and all patients provided signed informed consent.

### 2.2 Integrated diagnosis

The integrated diagnosis was performed using a list of 60 molecular markers (Table S1), which included CDKN2A/B, TERT, EGFR, and chromosome copy number variations. The markers were detected using next‐generation sequencing, polymerase chain reaction‐based assays, and fluorescence in situ hybridization methods. The diagnosis of each patient was determined by a multidisciplinary team consisting of a neuropathologist and neurosurgeon, based on the WHO 2016 and 2021 classification of central nervous system tumor.

### 2.3 Statistical methods

Descriptive statistics were used to summarize the clinical features. To compare subgroups, the student *t*‐test, Wilcoxon test, chi‐square test, and Fisher's precise test were used. Overall survival (OS) was defined as the interval between neurosurgery and death of all cause. Survival analysis was conducted using Kaplan–Meier methods and the log‐rank test, and the median OS and 95% confidence interval (CI) were calculated. The analysis was performed using R (4.2.1). The Sankey diagram illustrating the changes in diagnosis from the WHO 4th edition classification to the 5th edition was created using the “networkD3” package. The waterfall plot was generated using the “ComplexHeatmap” package.

## 3 RESULTS

### 3.1 Baseline characteristics of patients and changed integrated diagnosis

**FIGURE 1:**
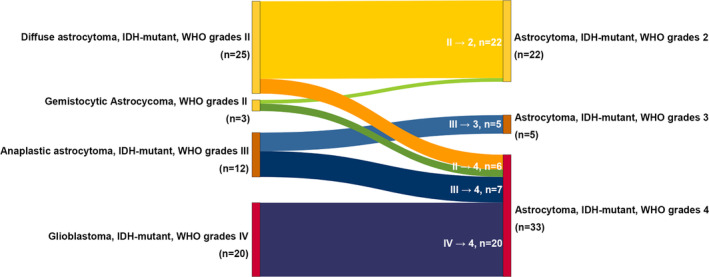
Diagnoses change from 2016 to 2021 classification.

This study included 60 patients diagnosed with astrocytoma, IDH‐mutant according to the WHO 2021 classification of tumors of central nervous system. The changed diagnoses were shown in Figure 1, based on the WHO 2016 and 2021 classifications. Of the 28 patients diagnosed with WHO grade II diffuse astrocytoma, six patients had their diagnosis changed to WHO grade 4 astrocytoma. Seven out of 12 patients had their diagnoses changed from WHO grade III anaplastic astrocytoma to WHO grade IV. All 20 patients with glioblastoma, IDH‐mutant, WHO grade IV were diagnosed with astrocytoma, IDH‐mutant, WHO grade IV.

The patients were divided into two groups: the astrocytoma, IDH‐mutant, WHO grade 2–3 group (*n* = 27) and WHO grade 4 group (*n* = 33), and baseline features were shown in Table 1. The gender ratio was 16:12 and 27:6 (*p* = 0.027), with an average age of 41 and 44 (*p* = 0.431), respectively. Twelve out of 33 patients in the WHO grade 4 group were recurrent astrocytoma, which were previously known as secondary glioblastoma. The most frequently observed clinical manifestations were related to intracranial hypertension and neurological function damage, such as headache, nausea, vomiting, and limb weakness etc. The median periods between detection of neurological symptoms and intracranial lesions to neurosurgery were 5 weeks and 6.5 weeks in WHO grade 2–3 group and WHO grade 4 group, respectively. More than half of the patients underwent gross total resection. Nine out of 27 patients (33.33%) and 15 out of 33 patients (45.45%) received a standard STUPP regime treatment, respectively.

**TABLE 1:** Baseline clinical characteristics of patients with IDH‐mutant astrocytoma.

	WHO 2–3 (*n* = 27)	WHO 4 (*n* = 33)	*p* Value
General features			
Sex			
Male	15 (55.56%)	27 (81.82%)	0.027*
Female	12 (44.44%)	6 (18.18%)	
Age (Mean ± SD, range)	40.85 ± 10.38, 21–62	44.12 ± 12.78, 23–74	0.431
BMI (Mean ± SD)	24.95 ± 3.14	23.82 ± 2.48	0.262
KPS (Median/range)	90 (10–100)	90 (10–100)	0.847
CCI (Median/range)	1 (0–3)	1 (0–7)	0.978
Detection‐to‐neurosurgery time/week (Median/range)	5 (1–676)	6.5 (1–832)	0.370
Recurrence	2 (7.41%)	12 (36.36%)	0.006*
Neurological symptoms			
Headache	13 (48.15%)	17 (51.52%)	0.467
Dizzy	6 (22.22%)	6 (18.18%)	
Nausea and vomit	4 (14.81%)	9 (27.27%)	
Limb weakness	6 (22.22%)	8 (24.24%)	
Sensory deficit	3 (11.11%)	5 (15.15%)	
Vision and visual field disorders	2 (7.41%)	1 (3.03%)	
Urinary and fecal incontinence	1 (3.70%)	1 (3.03%)	
Seizure	8 (29.63%)	15 (45.45%)	
Mental disorder and personality change	3 (11.11%)	1 (3.03%)	
Treatment			
Neurosurgery			
Gross total resection	15 (55.56%)	19 (57.58%)	0.823
Subtotal resection	3 (11.11%)	3 (9.09%)	
Partial resection	4 (14.81%)	6 (18.18%)	
Biopsy	2 (7.41%)	5 (15.15%)	
NA	3 (11.11%)	0 (0.00%)	
Adjuvant therapy			
STUPP	9 (33.33%)	15 (45.45%)	0.088
Neurosurgery	2 (7.41%)	2 (6.06%)	
Radiotherapy	1 (3.70%)	2 (6.06%)	
Temozolomide	3 (11.11%)	3 (9.09%)	
Anti‐angiogenic therapy	2 (7.41%)	3 (9.09%)	
Other	1 (3.70%)	4 (12.12%)	
NA	6 (22.22%)	9 (27.27%)	

Abbreviations: BMI, body mass index; CCI, Charlson comorbidity index; KPS, Karnofsky performance status; NA, loss of data.

Bold values means *p* value < 0.05

MRI features were also reviewed in Table 2. The majority of patients had a single lesion located in the frontal or temporal lobe. The average diameter of tumors was 4.49 cm for WHO grade 2–3 group and 4.73 cm for WHO grade 4 group. Although a WHO grade 4 astrocytoma with IDH‐mutation predisposed to enhanced lesions and peritumoral edema, no statistical significance was found between the two groups involved in this study. For astrocytoma with peritumoral edema detected on T2‐weighted sequence MRI, the average diameter of edema was 1.85 cm and 2.41 cm, respectively, with no significant difference between the two groups. The area of necrosis exhibited a more heterogeneous pattern in the WHO grade 4 group.

**TABLE 2:** MRI features of involved IDH‐mutant astrocytoma.

	WHO 2–3 (*n* = 23/27)	WHO 4 (*n* = 26/33)	*p* Value
Single lesion	23 (100.00%)	23 (88.46%)	0.237
Bilateral lesion(s)	1 (4.35%)	5 (19.23%)	0.194
Location			
Frontal lobe	22 (95.65%)	21 (80.77%)	0.670
Temporal lobe	7 (30.43%)	5 (19.23%)	0.362
Parietal lobe	1 (4.35%)	7 (26.92%)	0.052
Occipital lobe	1 (4.35%)	1 (3.85%)	0.882
Insular lobe	4 (17.39%)	2 (7.69%)	0.403
Functional areas	9 (39.13%)	7 (26.92%)	0.306
Diameter of tumor/cm (Mean ± SD)	4.49 ± 1.84	4.73 ± 1.40	0.622
T1			
High	0 (0.00%)	1 (3.85%)	0.623
Low	14 (60.87%)	17 (65.38%)	
Mixed	7 (30.43%)	7 (26.92%)	
NA	2 (8.70%)	1 (3.85%)	
T2			
High	12 (52.17%)	15 (57.69%)	0.698
Mixed	11 (47.83%)	11 (42.31%)	
Contrast‐T1			
Homogeneou and high‐ enhancement	0 (0.00%)	1 (3.85%)	0.171
Homogeneou and moderate‐enhancement	3 (13.04%)	2 (7.69%)	
Circumscribed‐enhancement	1 (4.35%)	7 (26.92%)	
Mixed‐ enhancement	5 (21.74%)	7 (26.92%)	
Non‐ enhancement	14 (60.87%)	8 (30.77%)	
NA	0 (0.00%)	1 (3.85%)	
Peritumoral edema	12 (52.17%)	19 (73.08%)	0.084
Diameter of Peritumoral edema/cm (Mean ± SD)	1.85 ± 0.96	2.41 ± 1.06	0.144
Necrosis	10 (43.48%)	13 (50.00%)	0.302
Diameter of necrosis/cm (Mean ± SD)	2.36 ± 0.94	2.01 ± 1.19	0.436
Gyriform infiltration on FLAIR sequence^a^	0/6 (0.00%)	0/11 (0.00%)	–

^a^ Six out of 27 patients in WHO grade 2–3 astrocytoma group and 11 out of 33 patients in WHO grade 2–3 astrocytoma group had MRI FLAIR sequence.

*Note*: *p <* 0.05.

### 3.2 Molecular alternation

**FIGURE 2:**
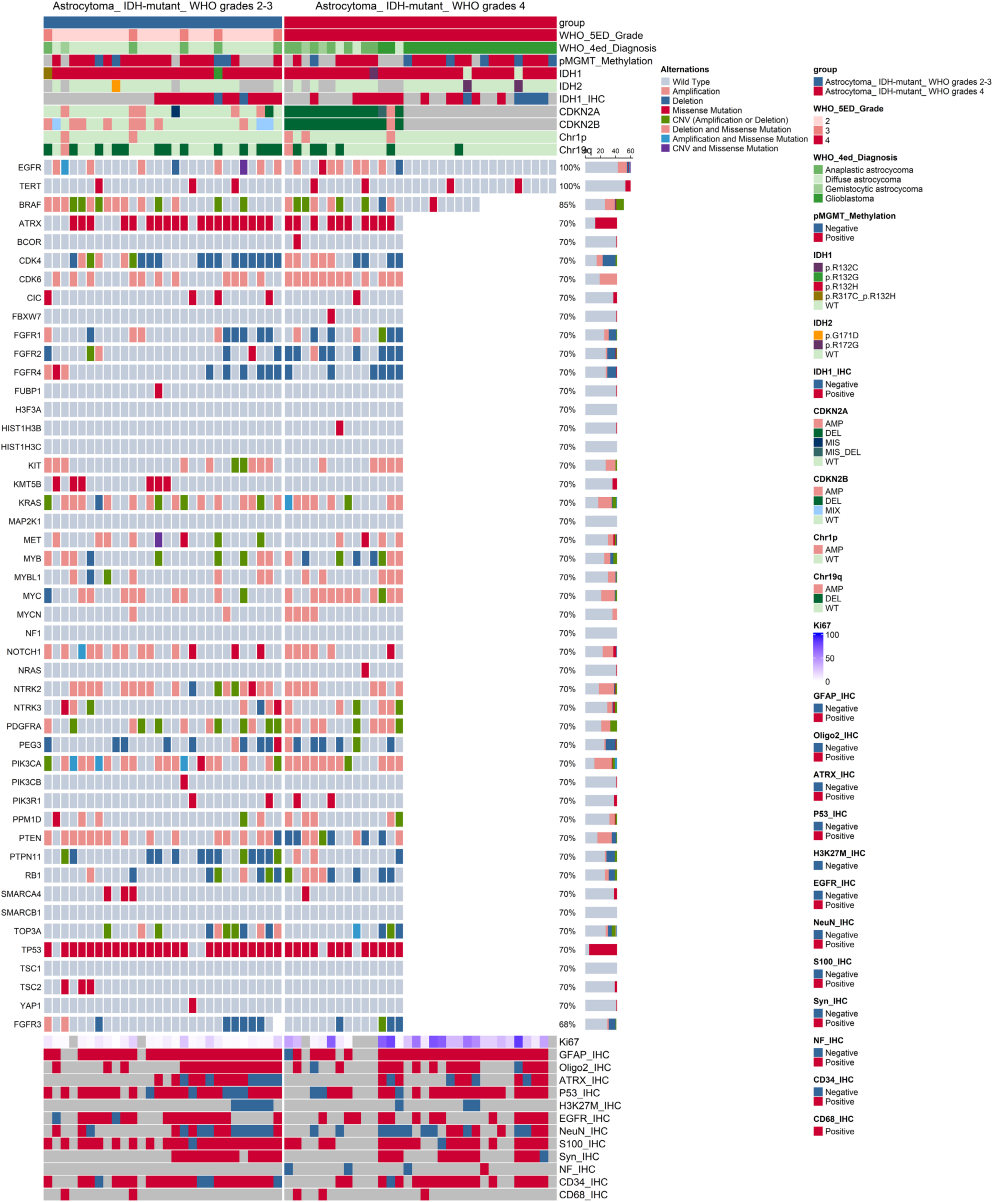
Pathological and molecular characteristic of patients with IDH‐mutant astrocytoma. The type of alternations was shown in the figure. And the label “IHC” is the abbreviation of “immunohistochemistry”.

The pattern of a series of pathological markers and a list of 60 molecular biomarkers was shown in Figure 2 and Table S2. The Ki‐67 index was significantly higher in the WHO grade 4 group (*p* < 0.001*).

Alterations of CDK6, CDKN2A, CDKN2B, FGFR2, and MYC were more likely to be detected in WHO grade 4 astrocytoma, while variations of chromosome 19q had a higher frequency in WHO grade 2–3 astrocytoma.

### 3.3 Survival analysis

**FIGURE 3:**
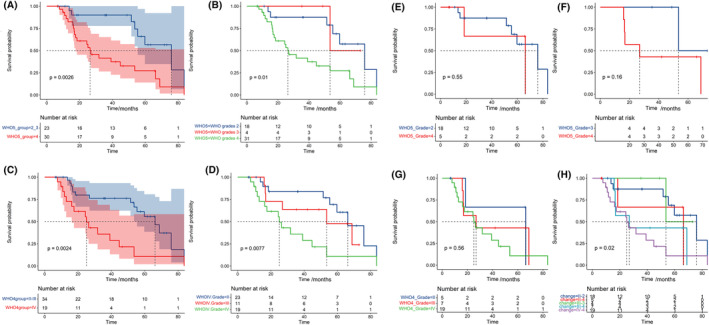
Kaplan–Meier curve of IDH‐mutant astrocytoma that diagnosed with 2016 and 2021 WHO classification of CNS tumors. The survival curve of patients with IDH‐mutant astrocytoma diagnosed with 2021 classification (A, B) and 2016 classification (C, D) was shown. (E) IDH‐mutant astrocytoma with grade II in 2016 but grade 2 and 4 in 5th edition classification. (F) IDH‐mutant astrocytoma with grade III in 2016 but grade 3 and 4 in 5th edition classification. (G) IDH‐mutant astrocytoma with grade II, III, and IV in 2016 but all as grade 4 in 5th edition classification. (G) Survival curve by the changes of tumor grades.

According to the WHO 5th edition of classification, the median OS of grade 2, 3, and 4 astrocytoma were 75.9 m, 53.6 m, and 26.4 m, respectively. In comparison, the WHO 4th edition of classification reported median OS for grade II, III, and IV astrocytoma as 66.2 m, 53.6 m, and 25.1 m, respectively (Figure 3). Further analysis of patients with changed diagnoses showed a shorter OS, particularly for those previously diagnosed with grade III. For patients diagnosed with grade II astrocytoma according to the WHO 4th edition classification, mOS was 75.9 months, which changed to 75.9 m for grades 2 and 66.2 m for grades 4 according to the WHO 5th edition classification. For patients diagnosed with grade III astrocytoma, the mOS was 53.6 months for grade 3 and 26.9 months for grade 4 according to the WHO 5th edition classification.

**FIGURE 4:**
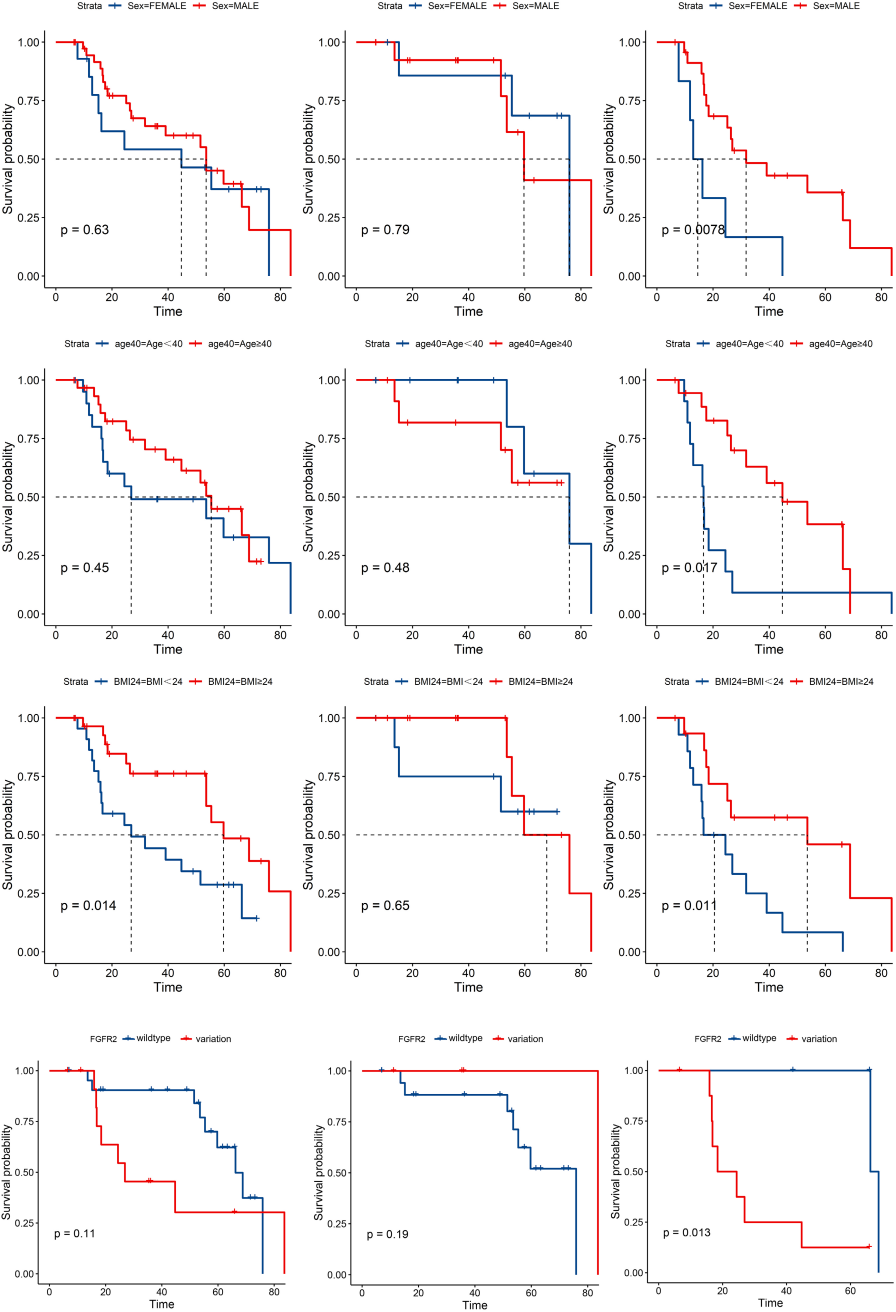
Kaplan–Meier curve of prognosis‐related factors. The first column was survival analysis in all patients involved, the second column was survival analysis in patients with WHO 2–3 grade astrocytoma, and the thrid column was survival analysis in patients with WHO 4 grade astrocytoma.

For univariate survival analysis, the median was used as the cutoff value for age, BMI, and other continuous variables. In the WHO grade 4 group, sex (*p* = 0.0078), age (*p* = 0.017), BMI (*p* = 0.011), and FGFR2 alternation (*p* = 0.013) had statistically significant results in univariate survival analysis (Figure 4). However, the univariate survival analysis of eloquent tumor, peritumoral edema in MRI, and gross total resection (GTR) did not have statistical significance, respectively (Figure S2). The correlation analysis between each factor was shown in Figure S1.

Variations of BCOR, MYCN, NOTCH1, and PIK3R1 and positive immunohistochemistry for S100 showed statistically significant results in univariate survival analysis in WHO grade 4 astrocytoma (Figure S2). In grade 2–3 astrocytoma, variations in FGFR4, KIT, NTRK2, and positive immunohistochemistry for ATRX and EGFR showed statistically significant results in univariate survival analysis (Figure S2).

## 4 DISCUSSION

Our study summarized the management experiences of astrocytoma with IDH‐mutation in our center. Patients whose diagnoses changed from WHO grade III in 2016 to WHO grade 4 in the 2021 classification had significantly worse overall survival.

No statistically significant difference in clinical and MRI features was found between WHO grade 2–3 and WHO grade 4 groups. However, patients with astrocytoma, WHO grade 4, showed a tendency to have peritumoral edema on MRI. In another retrospective study, the peritumoral zones on T2 phase of MRI were compared between glioblastoma and anaplastic astrocytoma. The study found that glioblastoma was associated with a higher decrease in signal intensity, both in IDH‐wildtype and IDH‐mutant subgroups. Mesny et al. report that gyriform infiltration is an imaging biomarker for molecular glioblastomas, and they did not find a “gyriform infiltration” pattern in IDH‐mutant astrocytoma.^8^ Gyriform infiltration refers to cortical hypersignal on MRI FLAIR sequence without enhancement on contrast sequence in IDH‐mutat astrocytoma. There was no difference in the size of peritumoral edema.^9^

Joyner et al found that the apparent diffusion coefficient (ADC) maps and cerebral blood volume (rCBV) maps were associated with the tumor grade of astrocytoma.^10^ Yang et al. established an IDH‐mutant astrocytoma group based on the 2021 classification and observed a lower minimum ADC value and a higher maximum rCBV value for grade 4 astrocytoma.^11^

Proton MR spectroscopy has recently demonstrated potential in distinguishing between subgroups of high‐grade diffuse astrocytoma. WHO grades 3 and 4 astrocytomas exhibited a significantly different myoinositol signal at short echo time.^12^

IDH‐mutant astrocytoma grade 4 group exhibited a higher frequency of alternations in FGFR2, CDK6, and MYC. The chromatin modifiers pathway and RTK/PI3K/AKT pathway were the two most frequent alternative pathways in IDH‐mutant primary astrocytoma, WHO grades 4, and FGFR2 alternations were detected in 6% of them.^13^ Although FGFR2 is a hot therapy target, downstream suppression of the PI3K pathway was found in several cases,^14^ making it an inefficient biomarker. IDH‐mutant astrocytoma with mutations in DNA mismatch repair genes have been reported to have a worse OS.^15^

According to the American Society of Clinical Oncology (ASCO) and the Society for Neuro‐Oncology (SNO) guidelines, radiotherapy and adjuvant temozolomide (TMZ) are recommended for astrocytoma, IDH‐mutant, WHO grade 3.^16^ However, there is a lack of evidence for this treatment regimen under the new classification of IDH‐mutant astrocytoma, WHO grade 4. A multicenter study has shown that patients with primary IDH‐mutant glioblastoma may benefit from TMZ and concurrent radiochemotherapy, leading to an overall survival benefit.^17^ In the progression of WHO 4 glioblastoma/astrocytoma, recurrent IDH‐mutant astrocytoma is less likely to extend beyond the gross tumor volume, which may require a modified plan of radiotherapy or radiochemotherapy.^18^ Previous studies have identified the MGMT promoter has been identified as a biomarker of TMZ in both IDH‐wildtype and IDH‐mutant glioblastoma.^19^ These findings may need validation under the 2021 classification.

Our work had limitations, including its retrospective nature, small sample size, and potential for informative bias. We only performed univariate survival for clinical features and genetics alternations. Firstly, we should notice that *p* < 0.05 did not always equal to a significant difference{Amrhein, 2019 #20}. We have labeled the *p* value in every survival curve. Secondly, due to the limited sample size, multivariate analyses were not conducted for survival analysis or differentiation between subgroups of astrocytoma. It is important to validate the results of survival analyses in the future. It should be noted that the reported differences between the WHO grade 2–3 group and the WHO grade 4 group should be interpreted cautiously in clinical practice and require validation in the future. Furthermore, the study lacks details on MRI and genomic alterations, such as a quantitative analysis of ADC maps. We did not provide further information on patients with pathological grade 4 astrocytoma, specifically IDH‐mutant glioblastoma, or patients with molecular grade 4 astrocytoma with CDKN2A/B homozygous deletion. Furthermore, survival analyses to compare treatments between subgroups were not conducted. Additional research is needed on the biological multi‐omics and radiomics of CNS tumors under the 2021 classification to modify diagnostic criteria and prognostic biomarker patterns. It is imperative to promptly clarify the management framework for clinical practice.

## AUTHOR CONTRIBUTIONS

**Yuekun Wang:** Conceptualization (equal); data curation (equal); formal analysis (lead); methodology (lead); project administration (lead); writing – original draft (lead). **Hao Xing:** Data curation (equal). **Xiaopeng Guo:** Conceptualization (equal); data curation (equal). **Wenlin Chen:** Conceptualization (equal); data curation (equal). **Yaning Wang:** Data curation (equal). **Tingyu Liang:** Data curation (equal). **Hai Wang:** Data curation (equal). **Yilin Li:** Data curation (equal). **Shanmu Jin:** Data curation (equal). **Yixin Shi:** Data curation (equal). **Delin Liu:** Data curation (equal). **Tianrui Yang:** Data curation (equal). **Yu Xia:** Data curation (equal). **Junlin Li:** Data curation (equal). **Jiaming Wu:** Data curation (equal). **Qianshu Liu:** Data curation (equal). **Tian Qu:** Data curation (equal). **Siying Guo:** Data curation (equal). **Huanzhang Li:** Data curation (equal). **Kun Zhang:** Data curation (equal). **Yu Wang:** Conceptualization (equal); writing – review and editing (supporting). **Wenbin Ma:** Conceptualization (lead); writing – review and editing (equal).

## FUNDING INFORMATION

This work was funded by the the National High Level Hospital Clinical Research Funding (2022‐PUMCH‐B‐113), the Tsinghua University‐Peking Union Medical College Hospital Initiative Scientific Research Program (2019ZLH101) and the Beijing Municipal Natural Science Foundation (19JCZDJC64200[Z]) for Wenbin Ma and Beijing Municipal Natural Science Foundation (7202150) and the National High Level Hospital Clinical Research Funding (2022‐PUMCH‐A‐019) for Yu Wang.

## CONTRIBUTION TO THE FIELD STATEMENT

Our study firstly described the clinical features, qualitative features of MRI and gene alternations of IDH‐mutant astrocytoma under 2021 classification of CNS tumors through our single‐center retrospective cohort. The pattern of peritumoral edema and gene various of PI3K pathway may be a potential marker of management of WHO grade 4 astrocytoma.

## Supporting information


Figure S1.

Figure S2.

Table S1.

Table S2.

## Data Availability

The data are available from the corresponding author on reasonable request.
