# Supplementary material for: Clinical features, MRI, molecular alternations, and prognosis of astrocytoma based on WHO 2021 classification of central nervous system tumors: A single‐center retrospective study
Source: Cancer Med. 2024 Jul 5;13(13):e7369. doi: 10.1002/cam4.7369 (PMC11226410; doi:10.1002/cam4.7369)
Supplement: Supplementary file 1 — Figure S1. [file CAM4-13-e7369-s001.docx]

Supplementary figure 1 Correlaship between clinical features and molecular markers


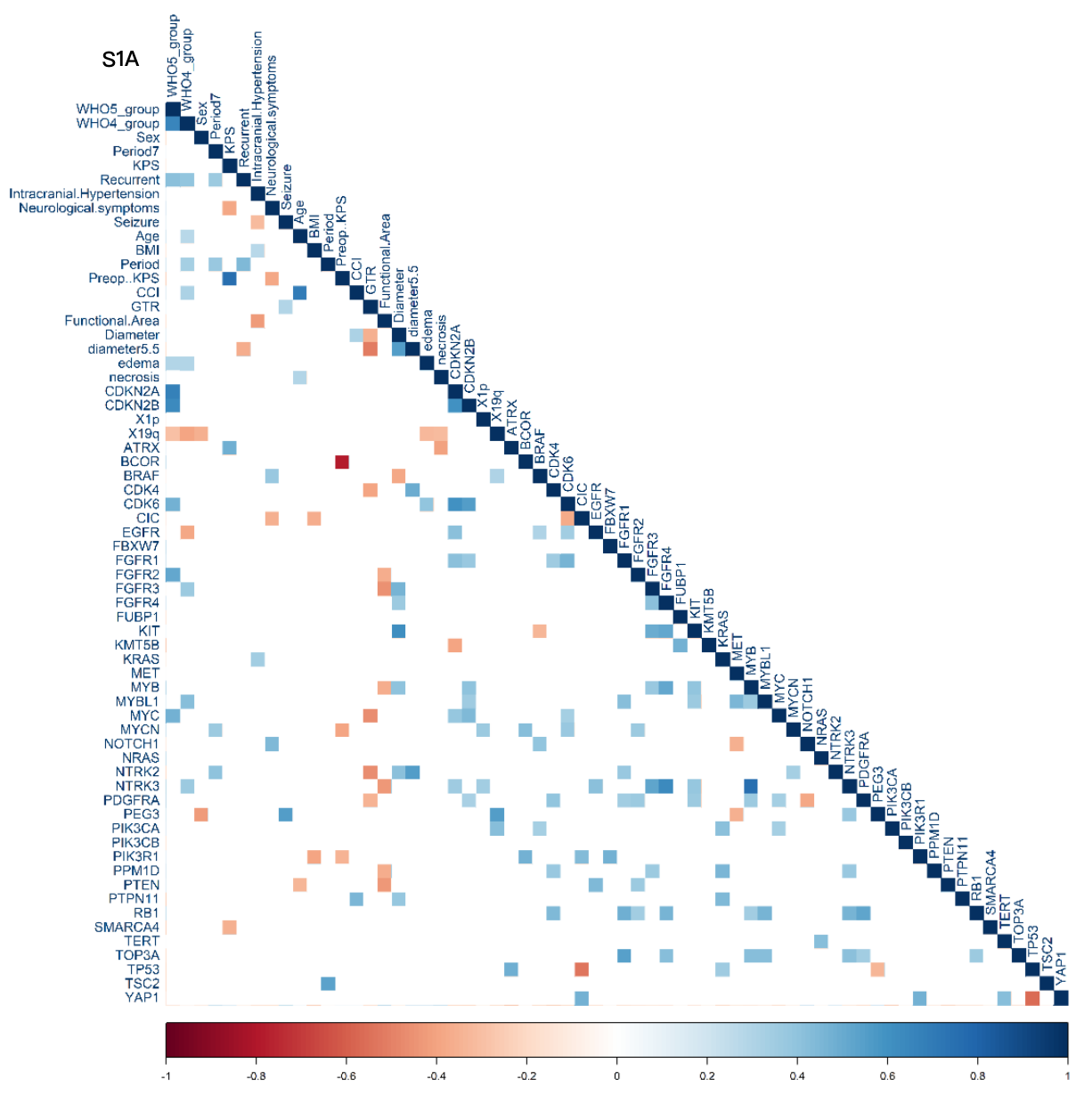

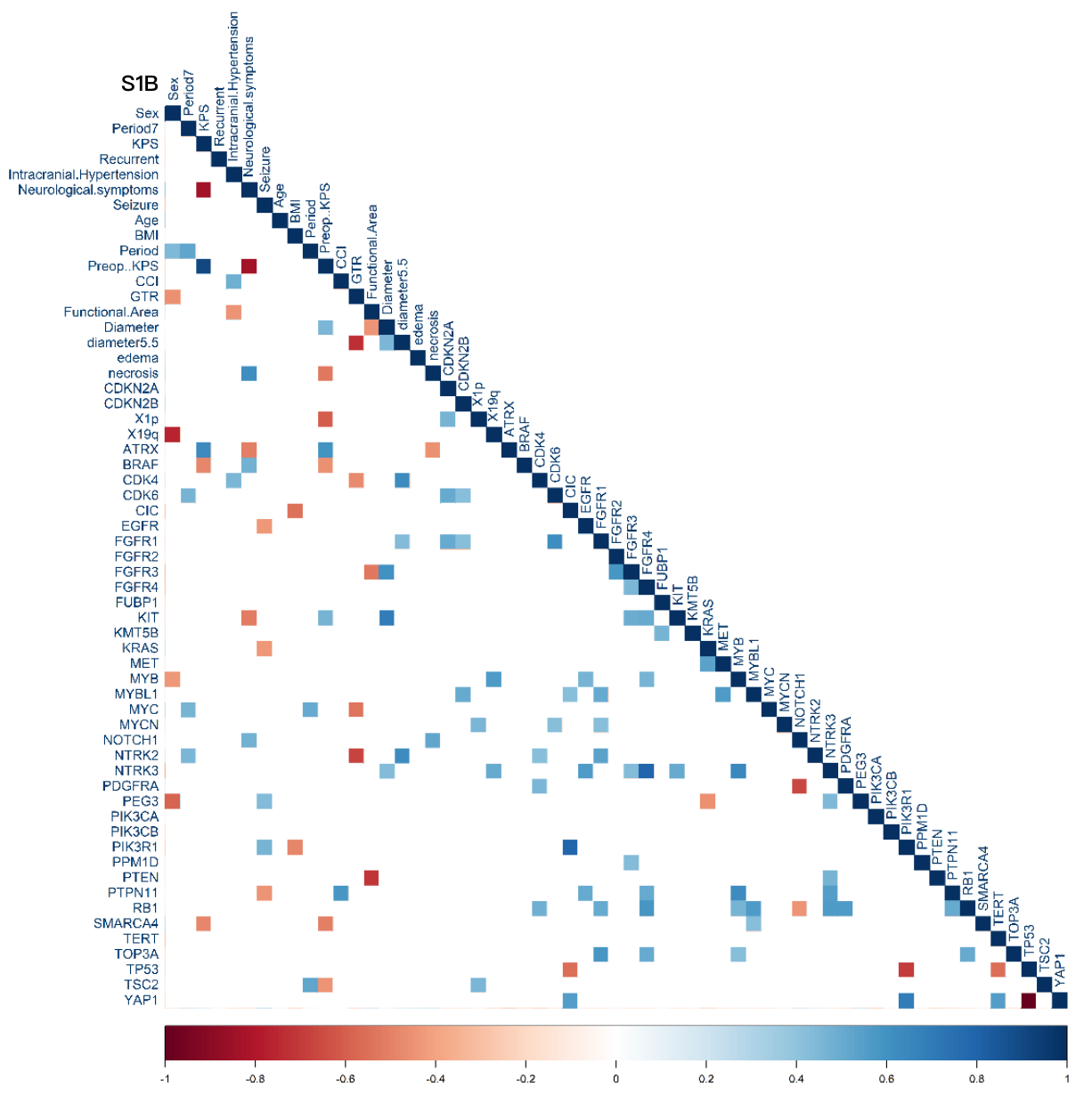

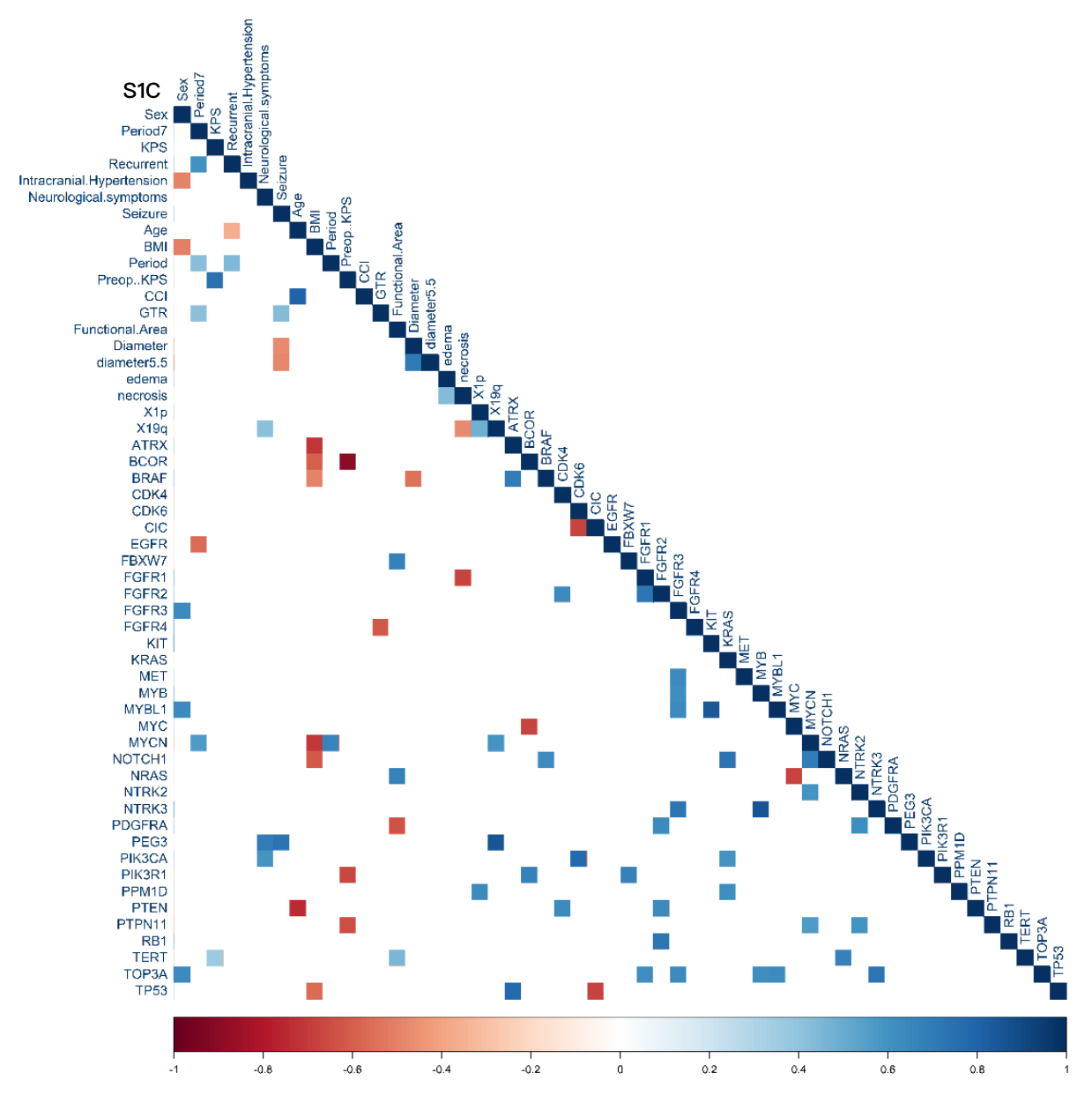


S1A, Correlaship between each factors among all patients.

S1B, Correlaship between each factors among WHO grade 2-3 group.

S1C, Correlaship between each factors among WHO grade 4 group.

Correlation coefficients were shown in squares and colorized referrence bar was shown below the matrix. Statistically significant result was shown in red and blue squares, and statistically non-significant results were shown in white squares.
