# Supplementary material for: Clinical features, MRI, molecular alternations, and prognosis of astrocytoma based on WHO 2021 classification of central nervous system tumors: A single‐center retrospective study
Source: Cancer Med. 2024 Jul 5;13(13):e7369. doi: 10.1002/cam4.7369 (PMC11226410; doi:10.1002/cam4.7369)
Supplement: Supplementary file 2 — Figure S2. [file CAM4-13-e7369-s002.docx]

Supplementary figure 2 Survival curve of clincial features and molecular markers


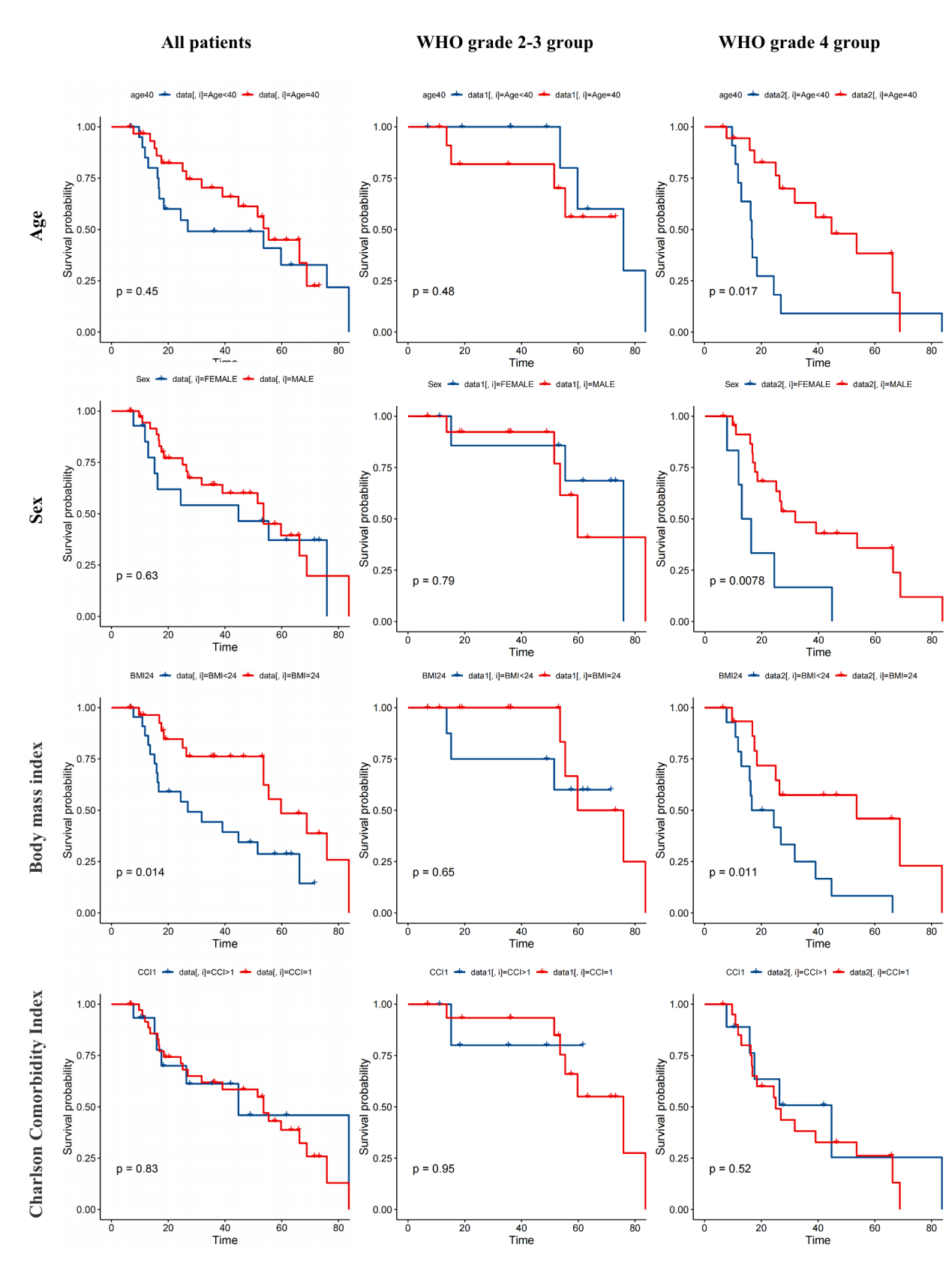

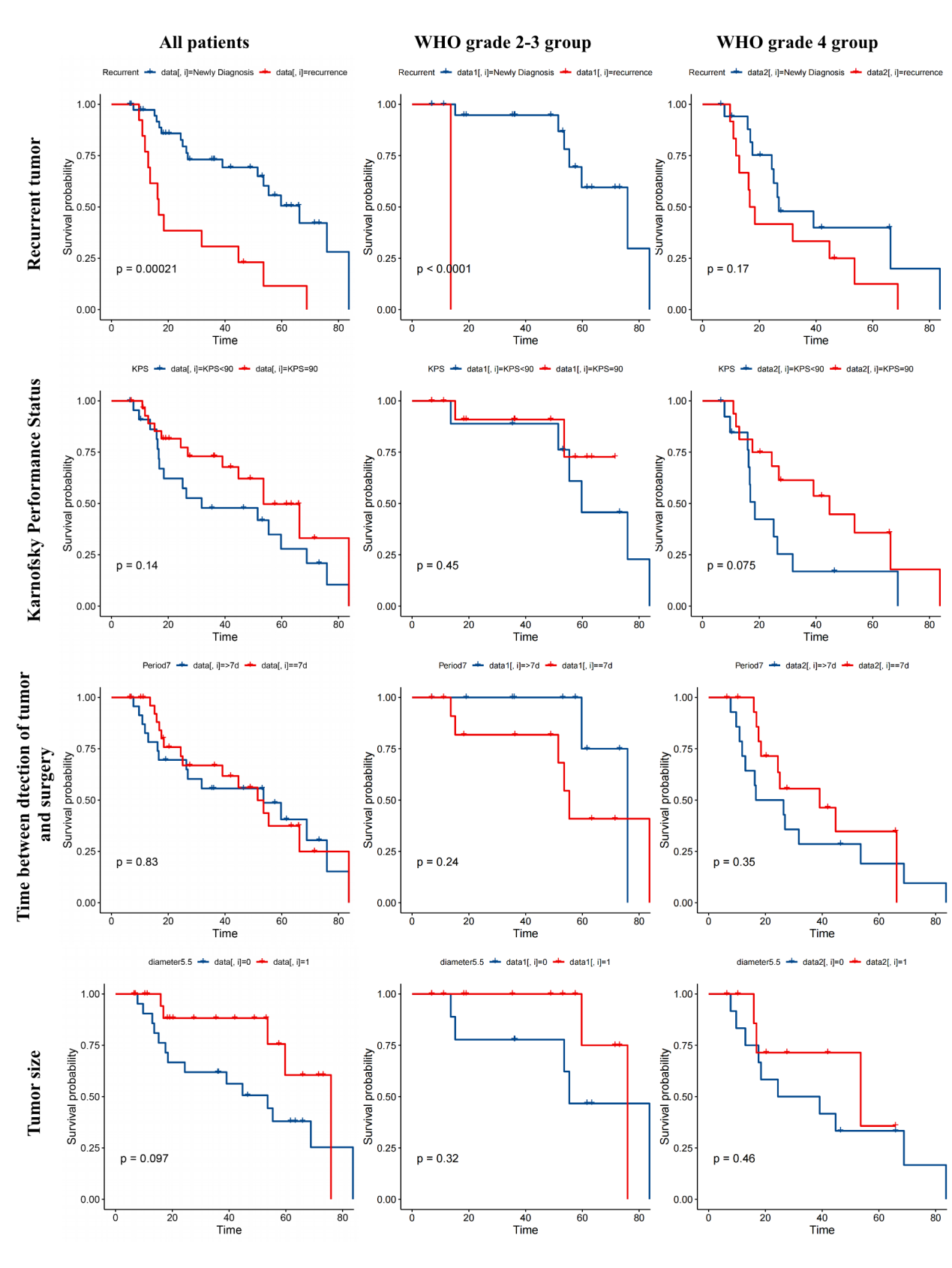

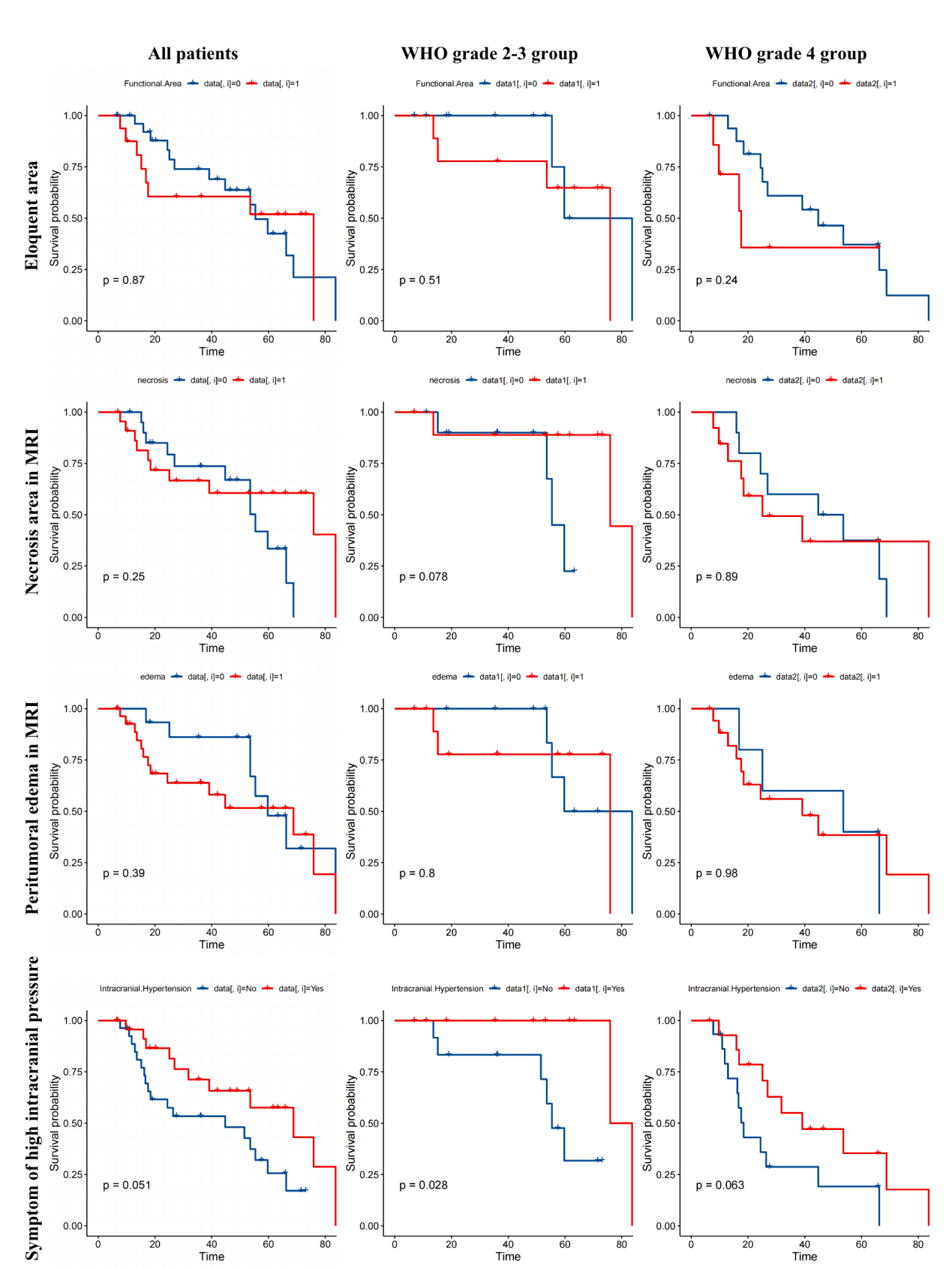

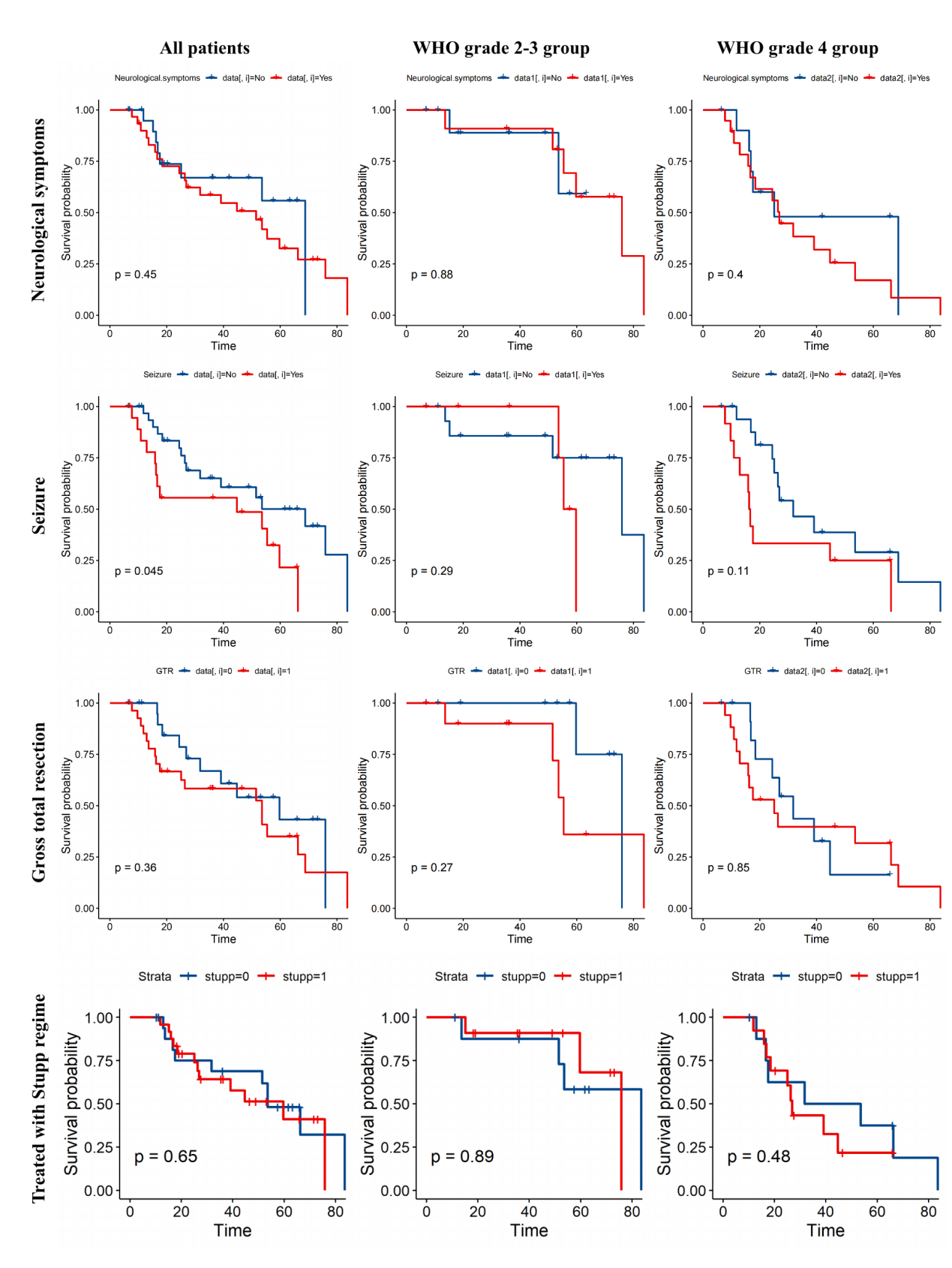

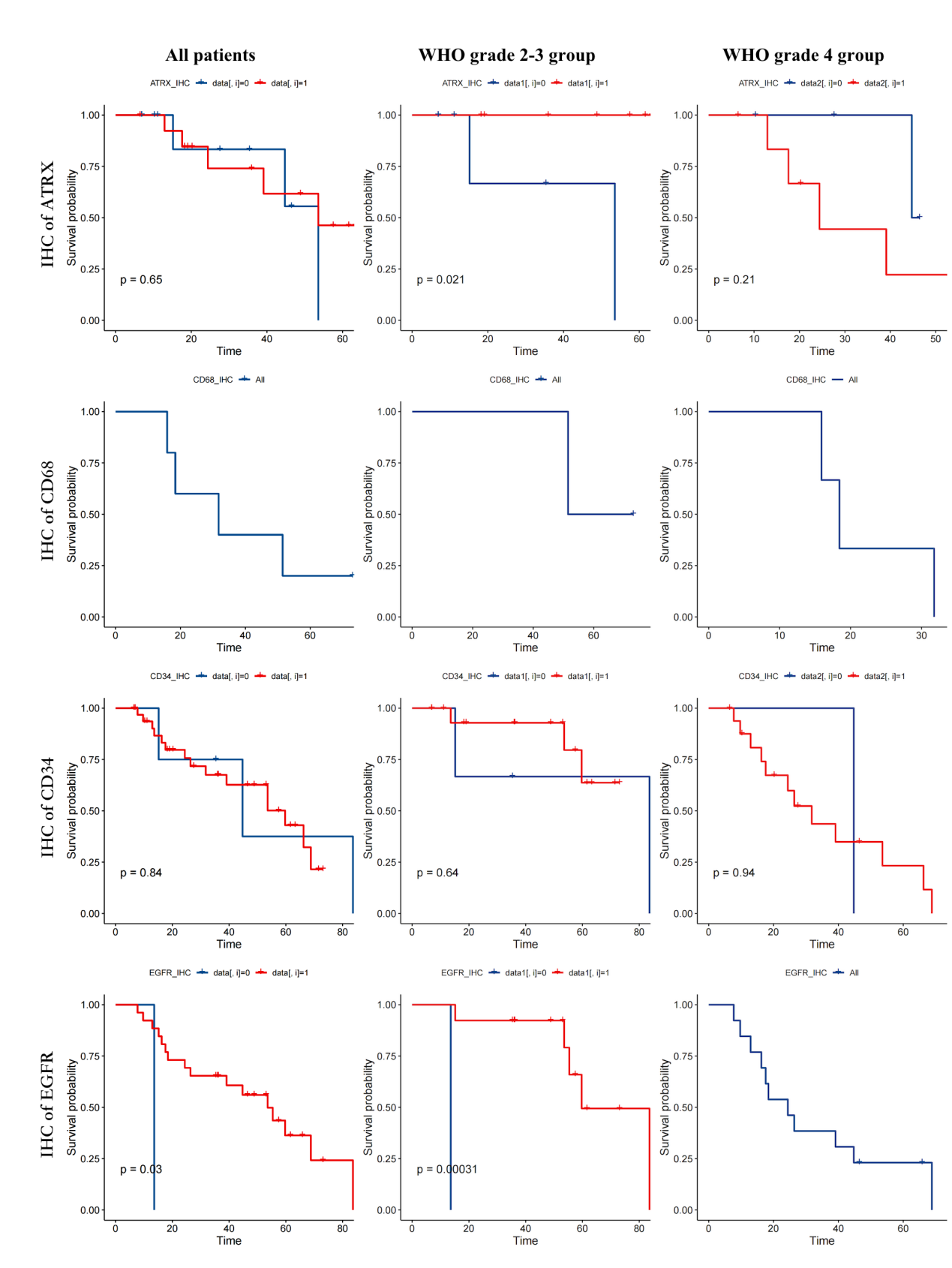

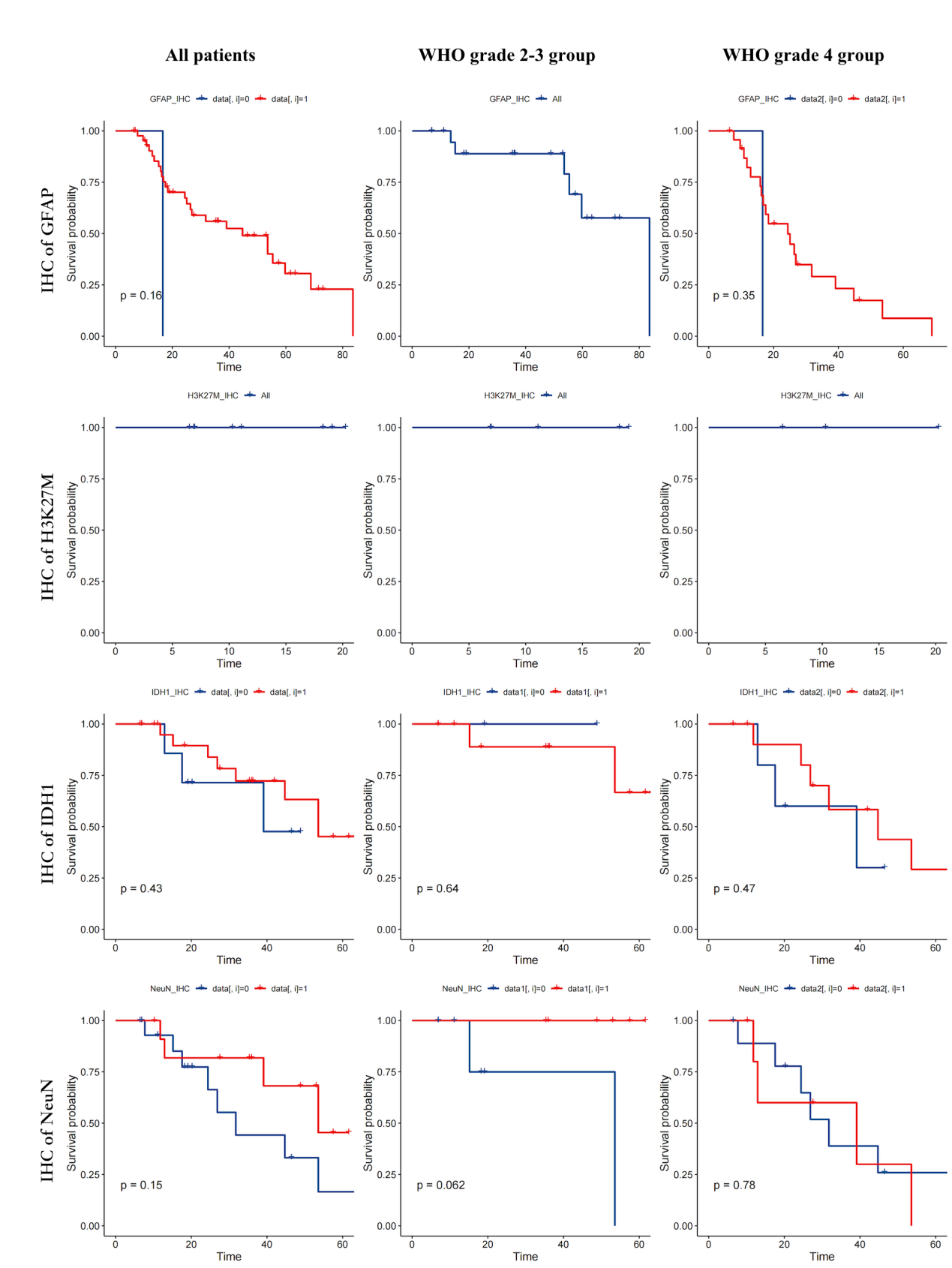

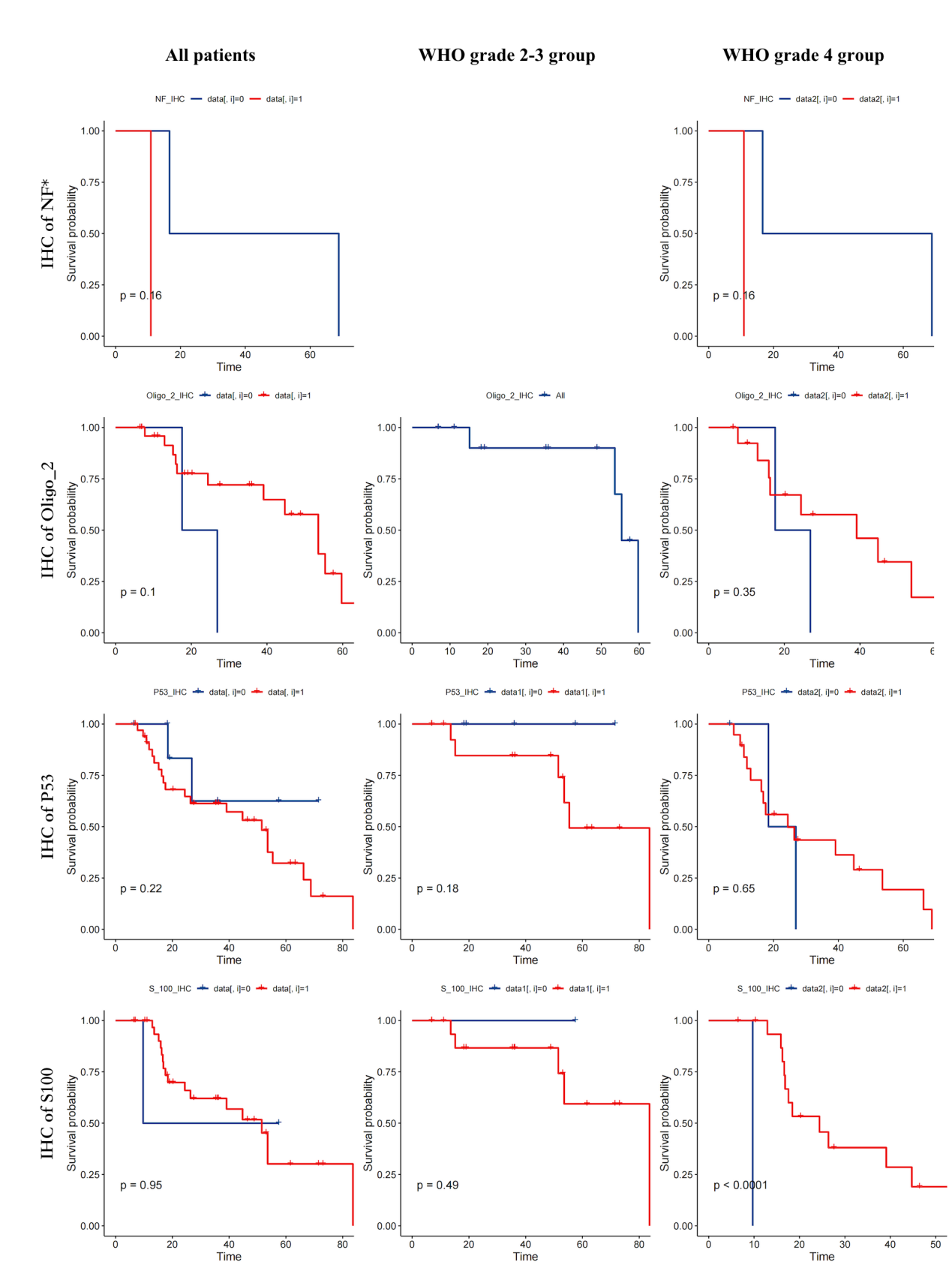

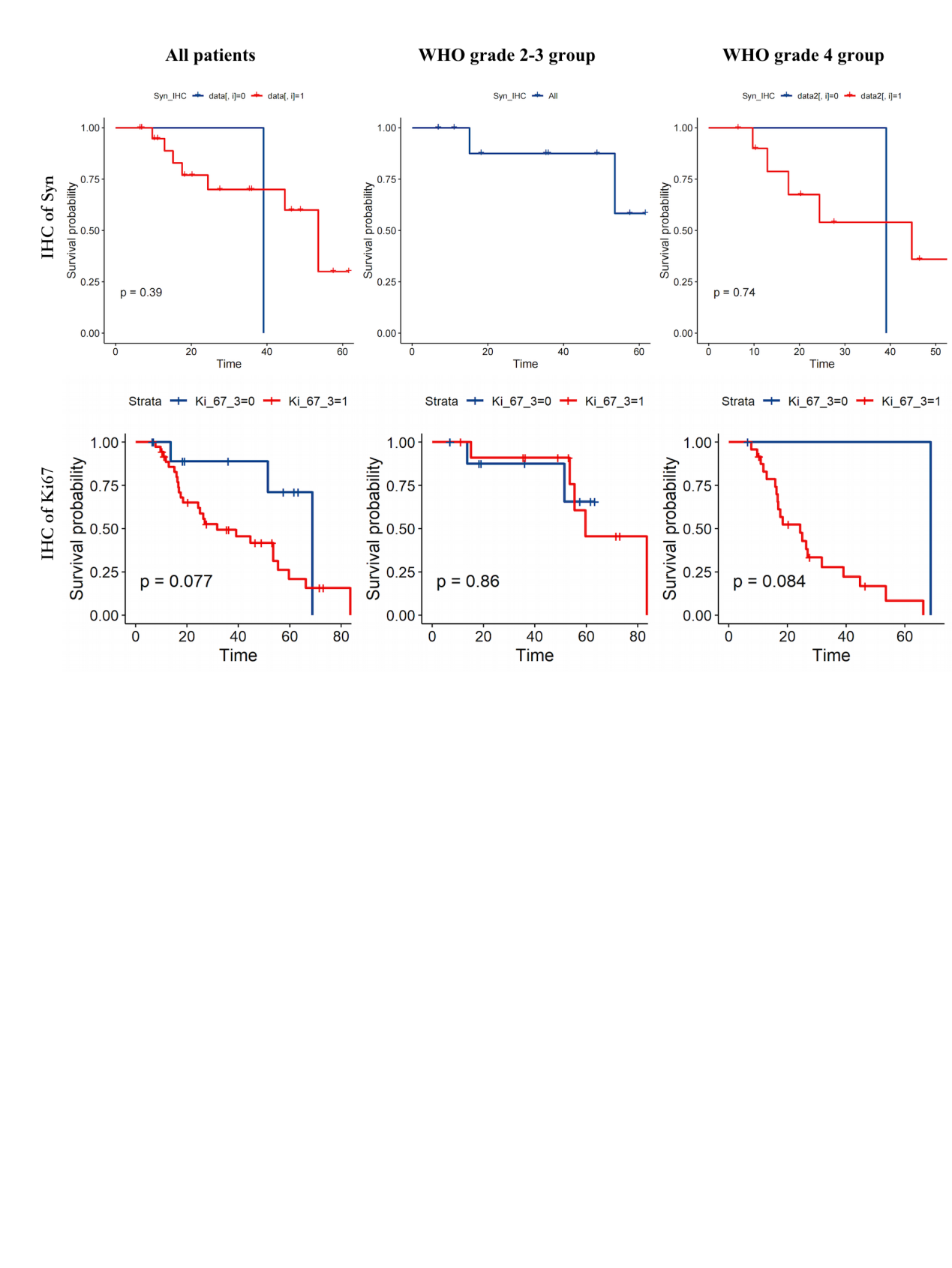

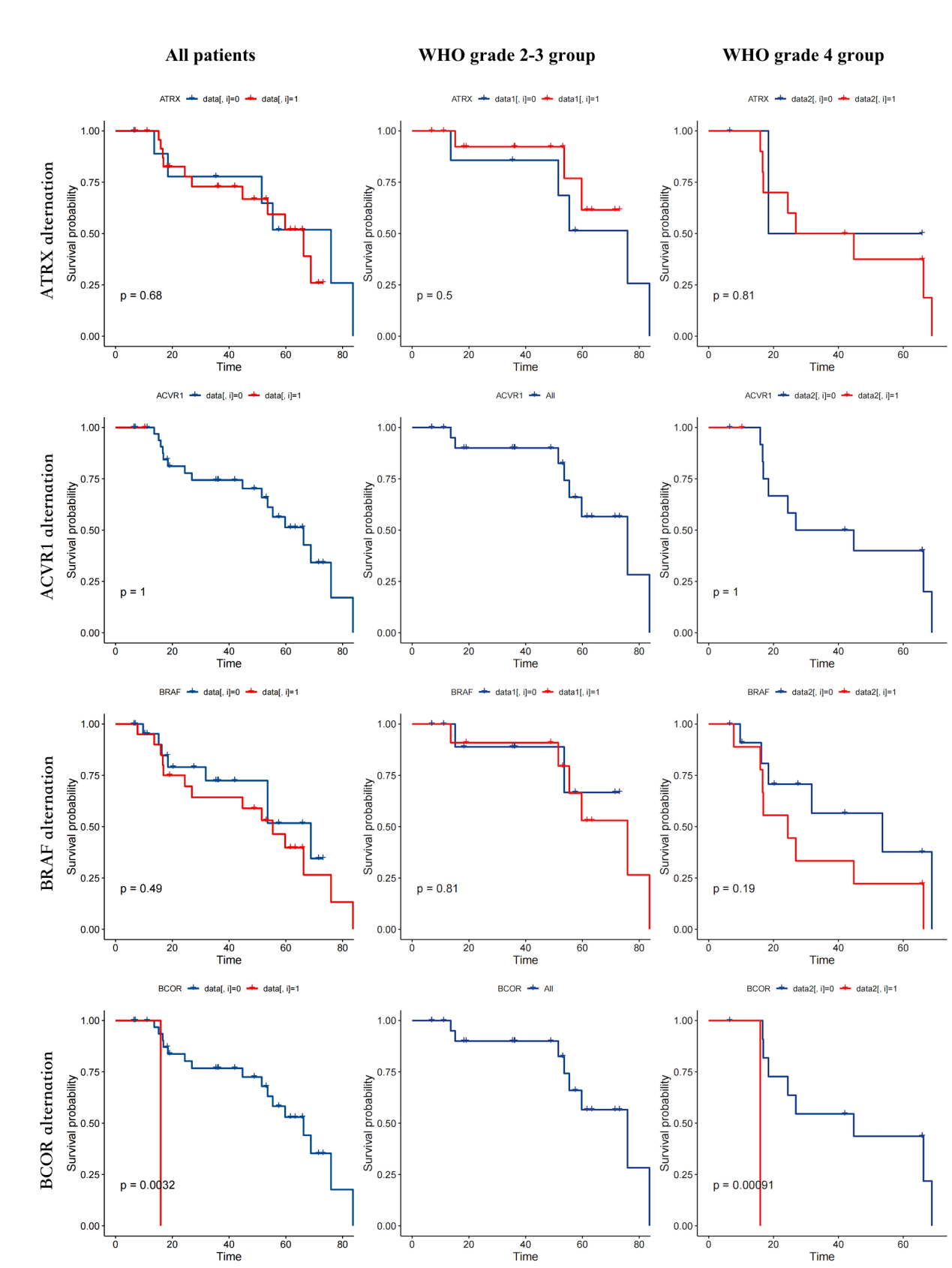

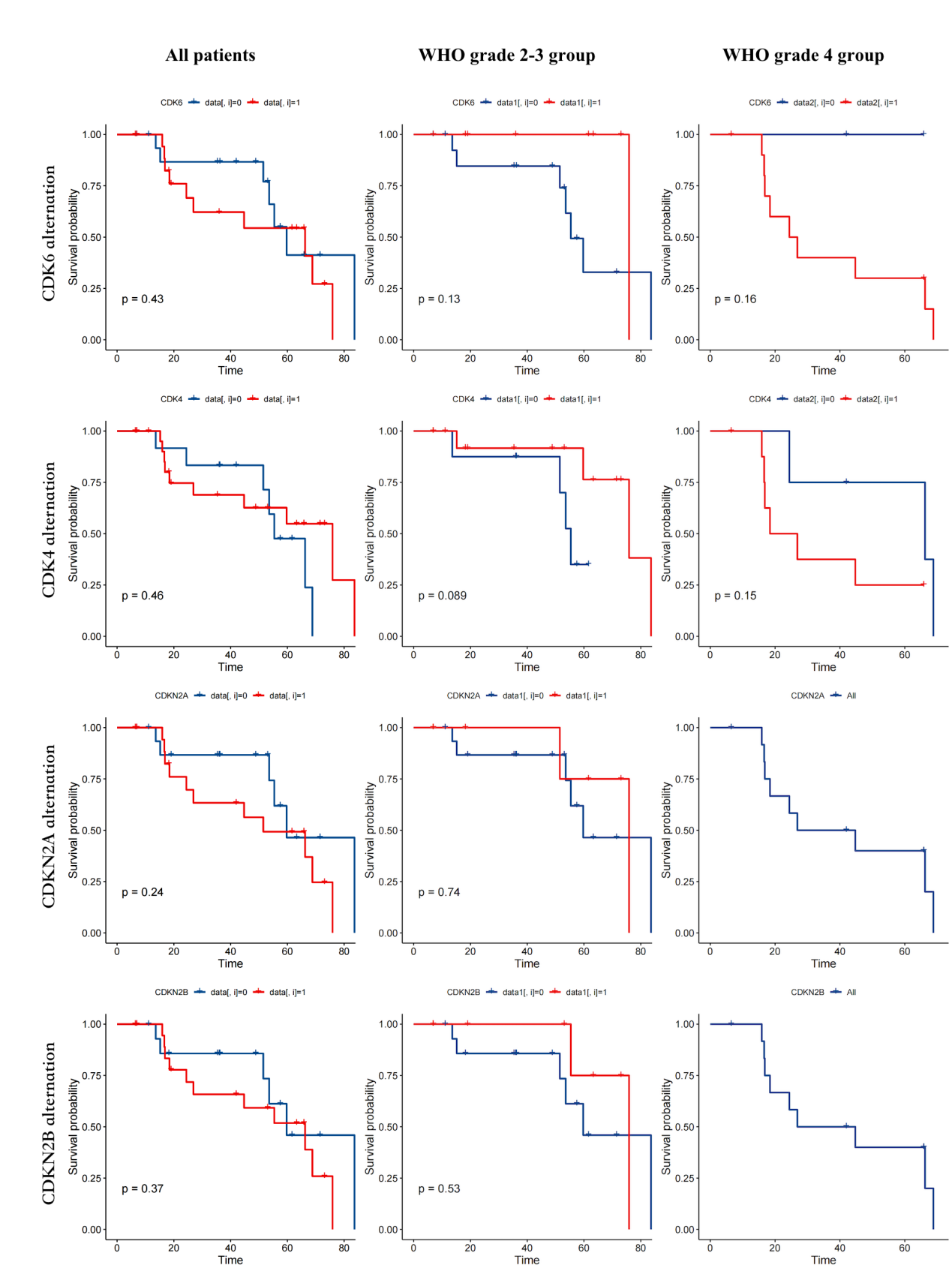

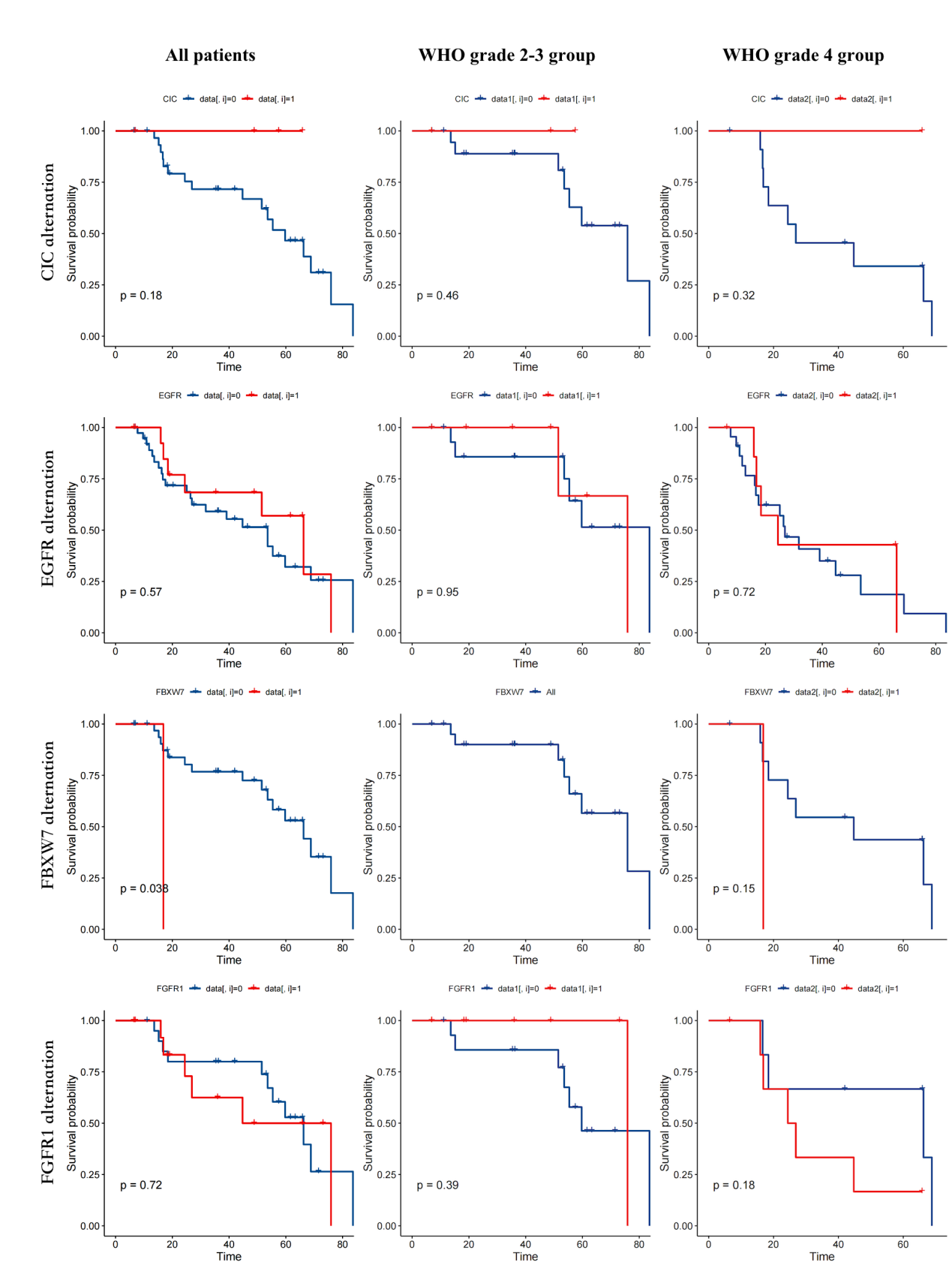

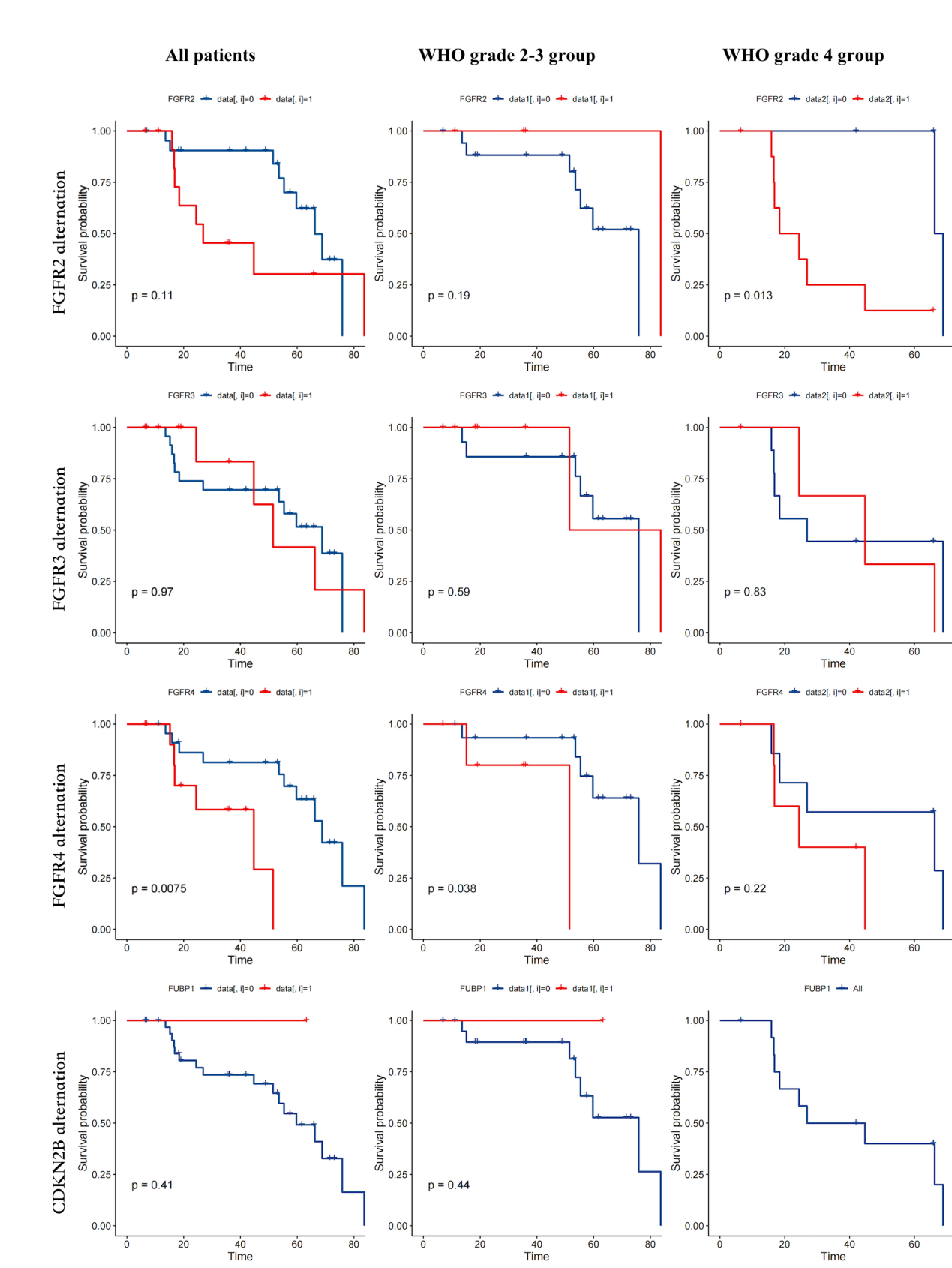

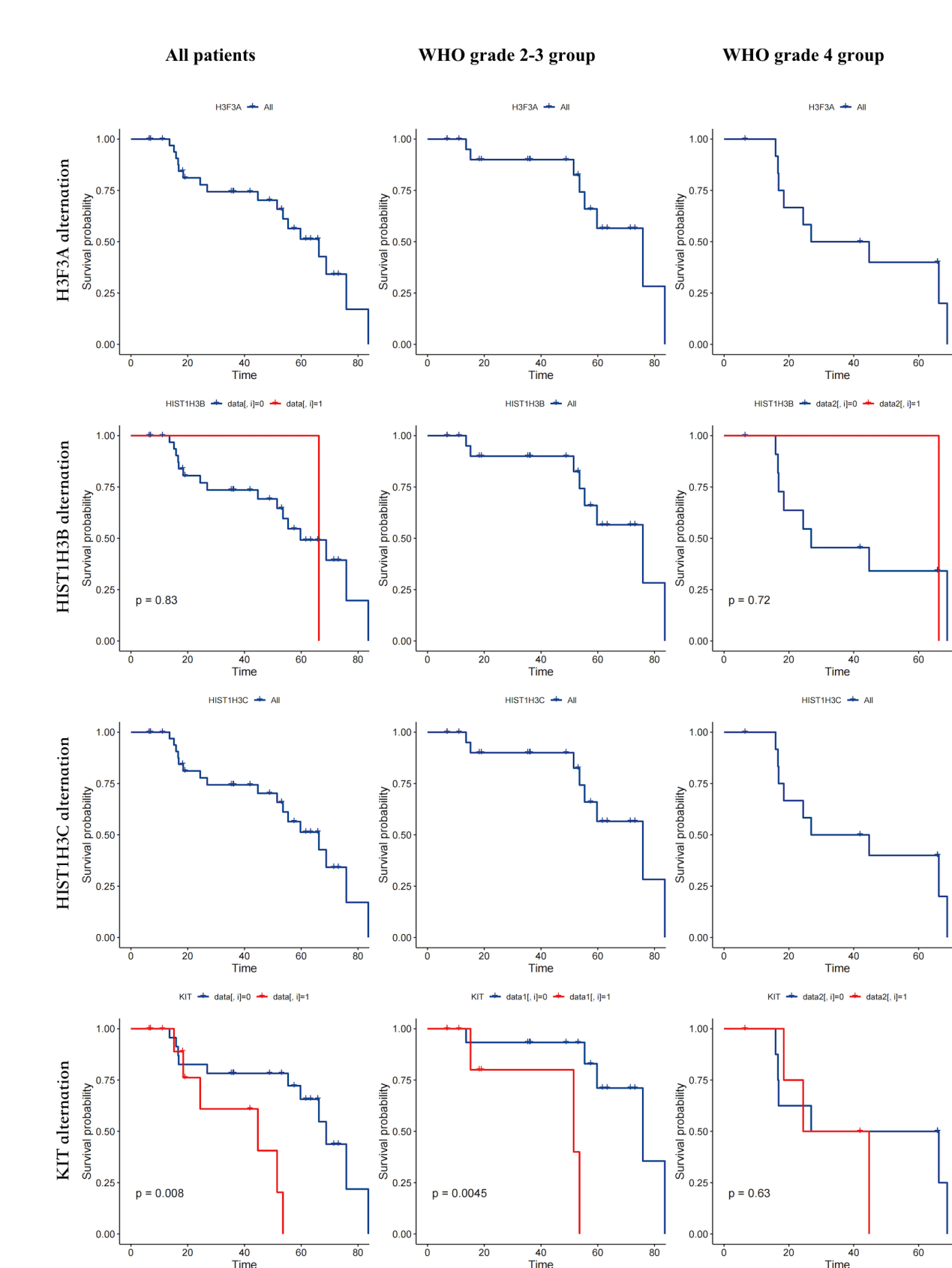

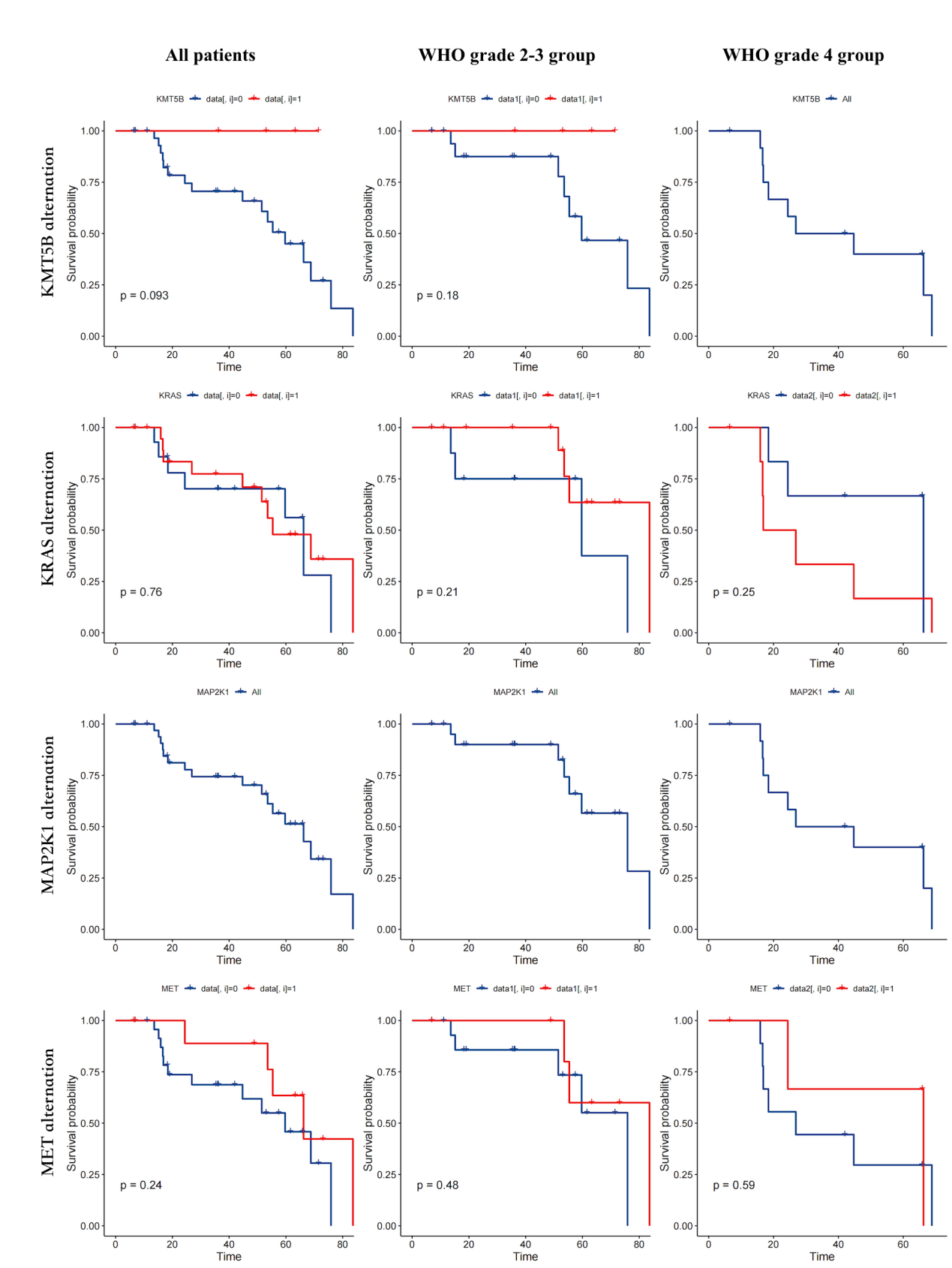

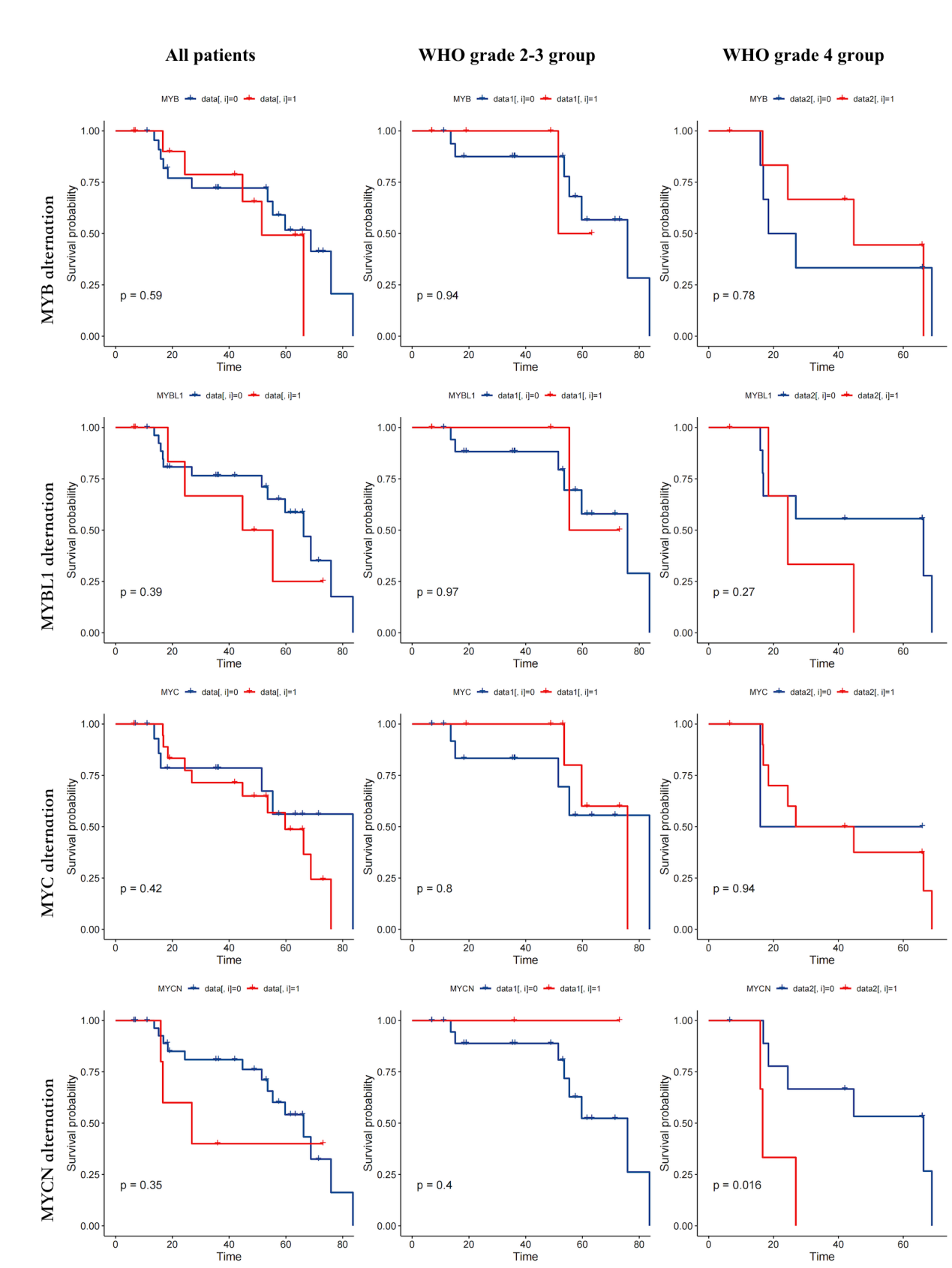

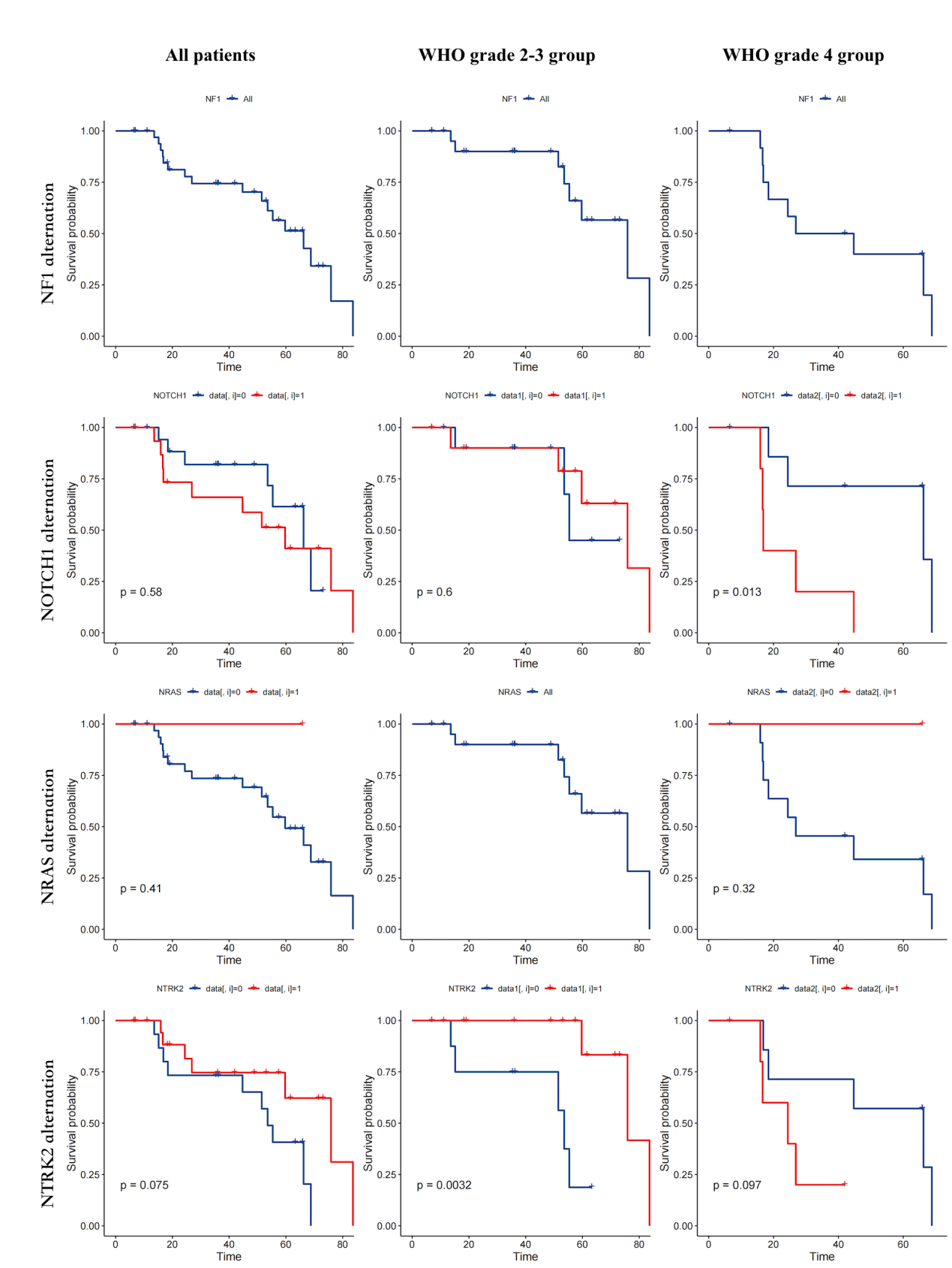

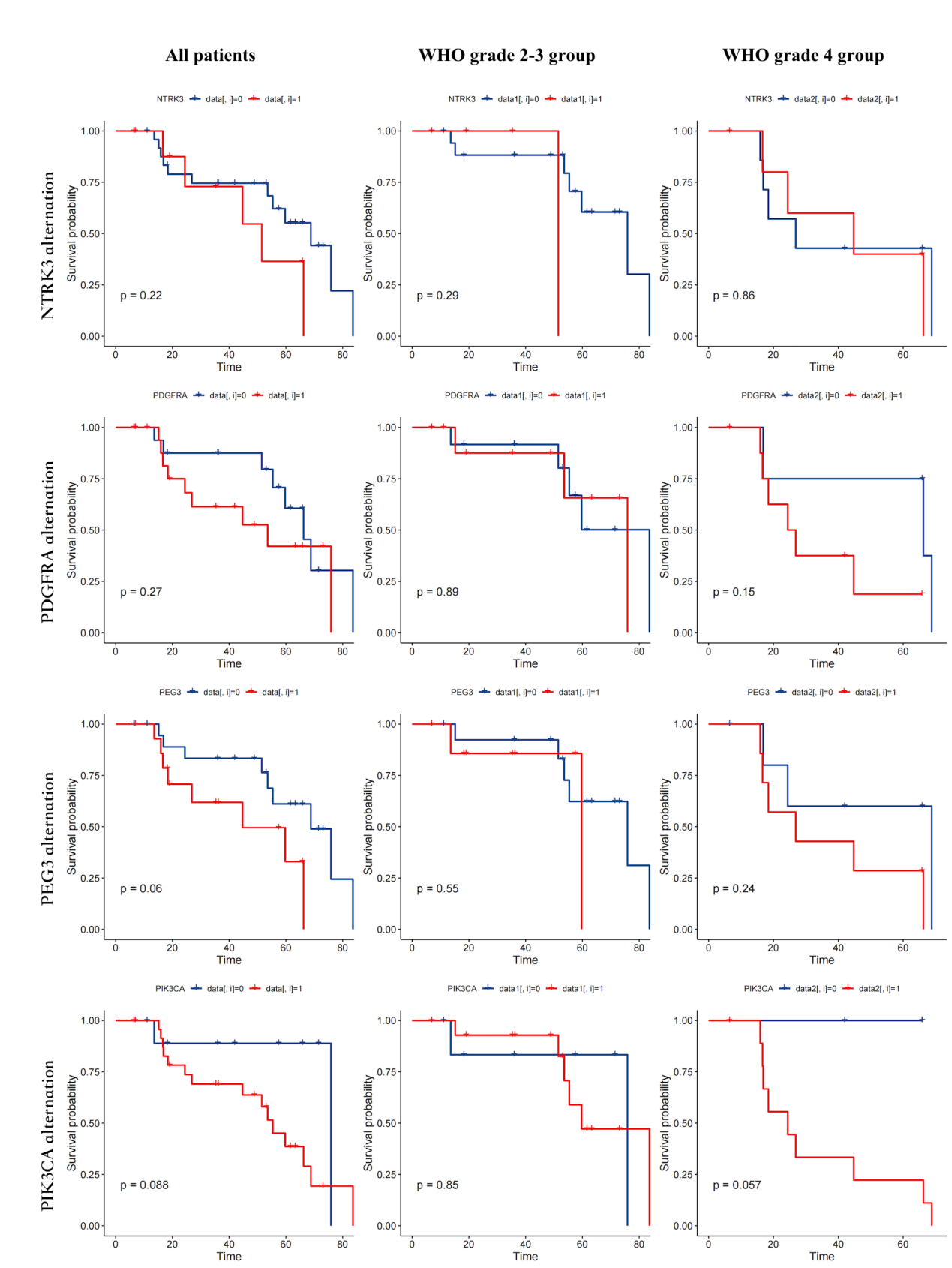

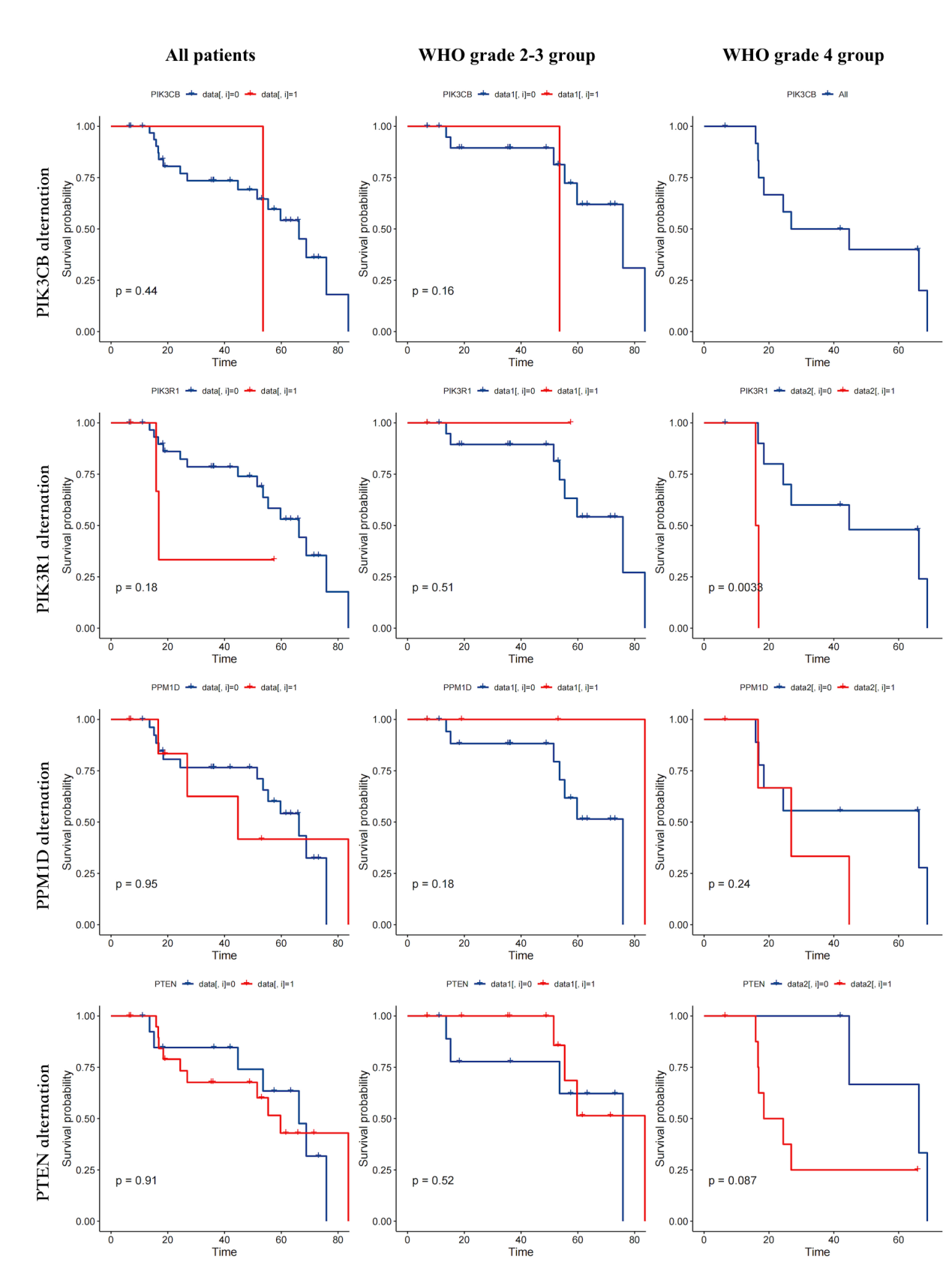

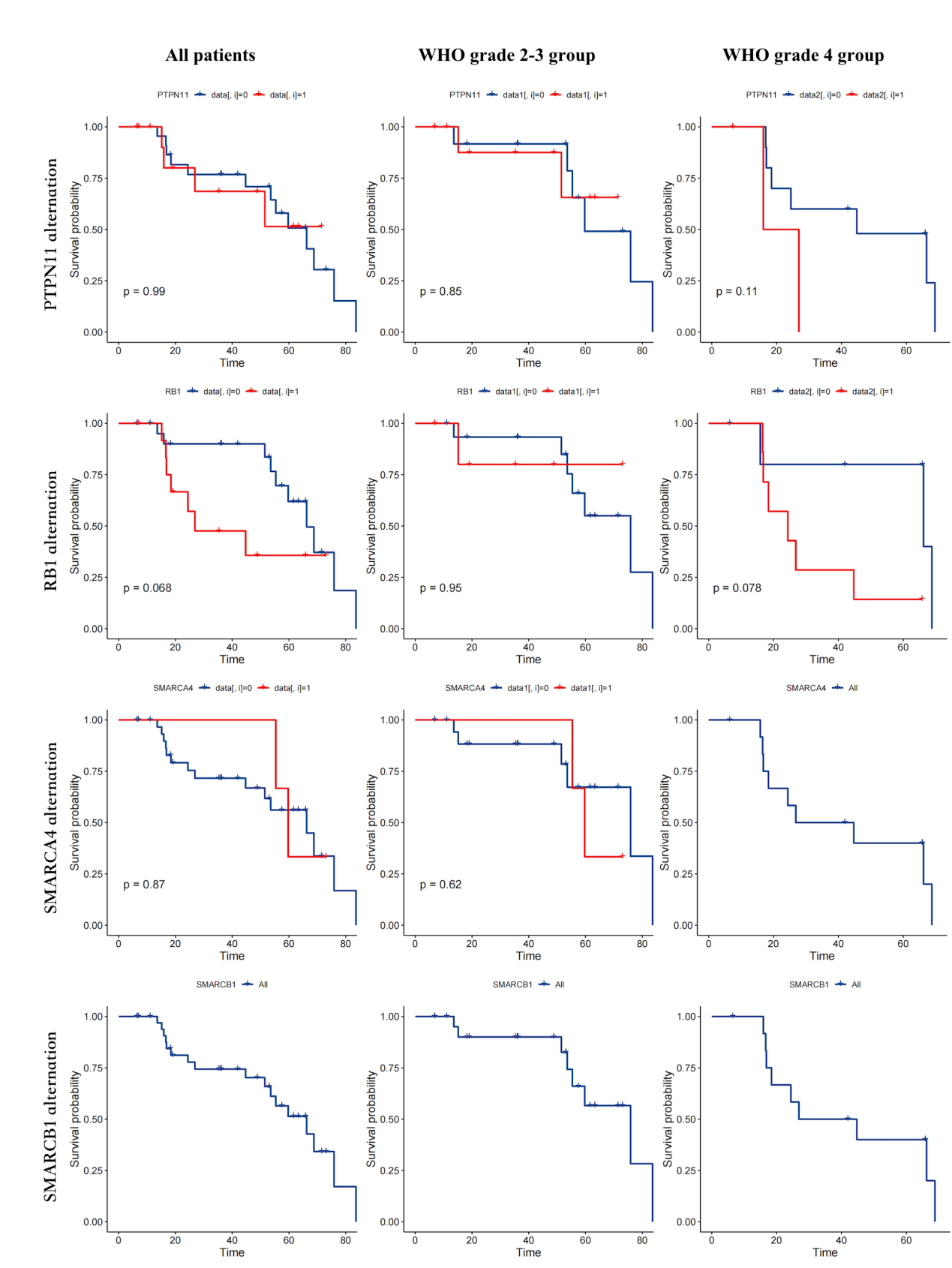

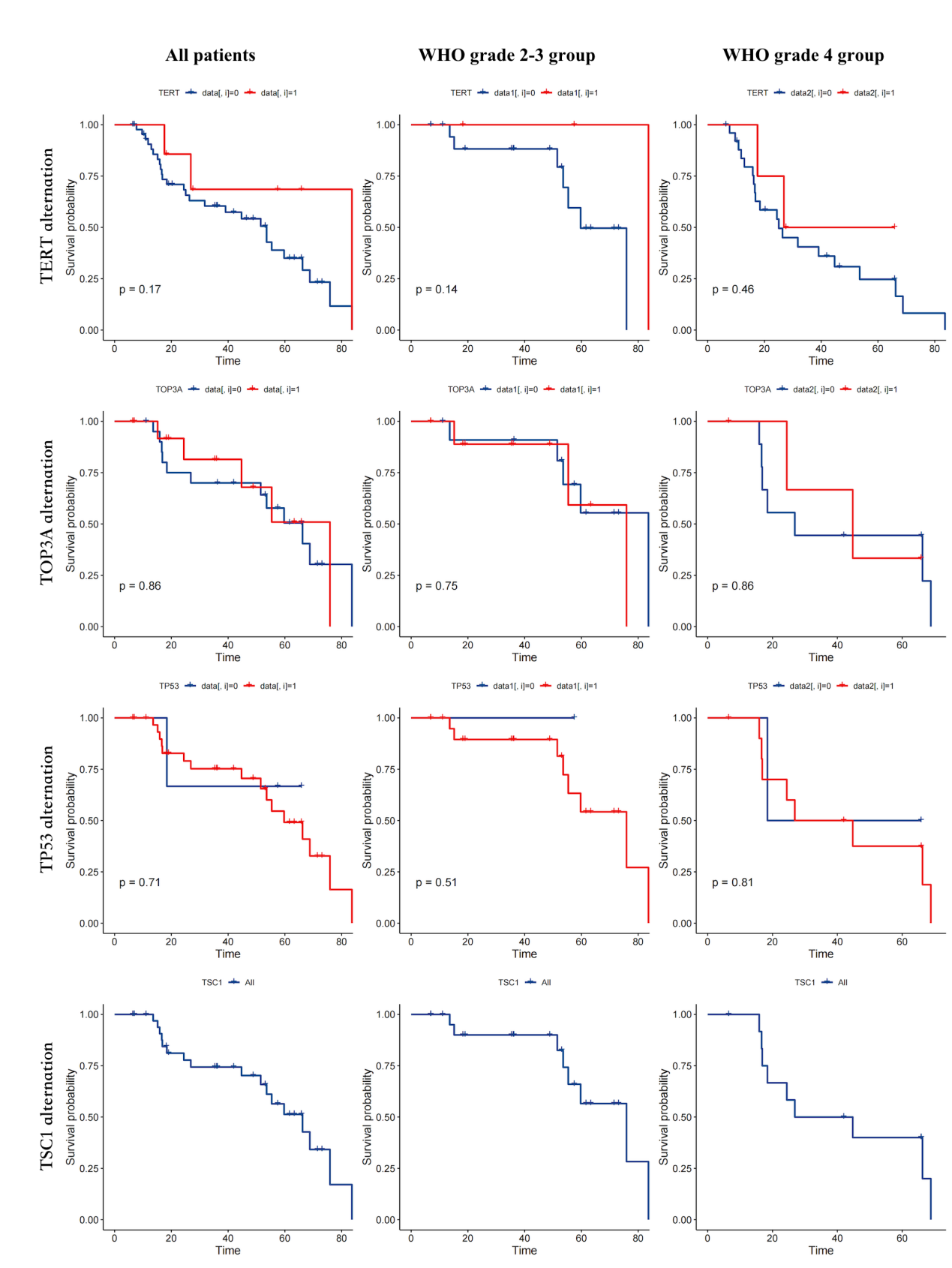

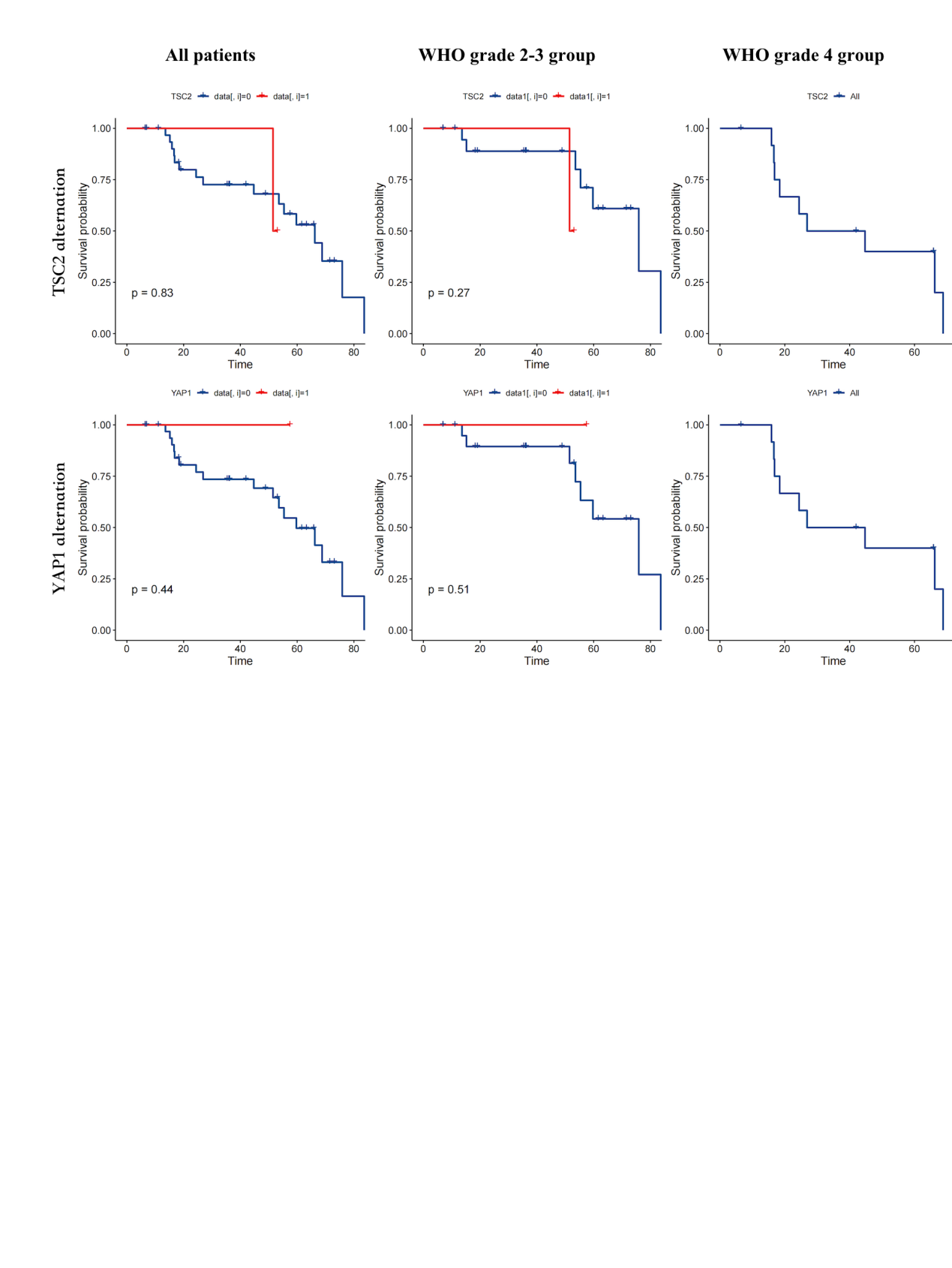

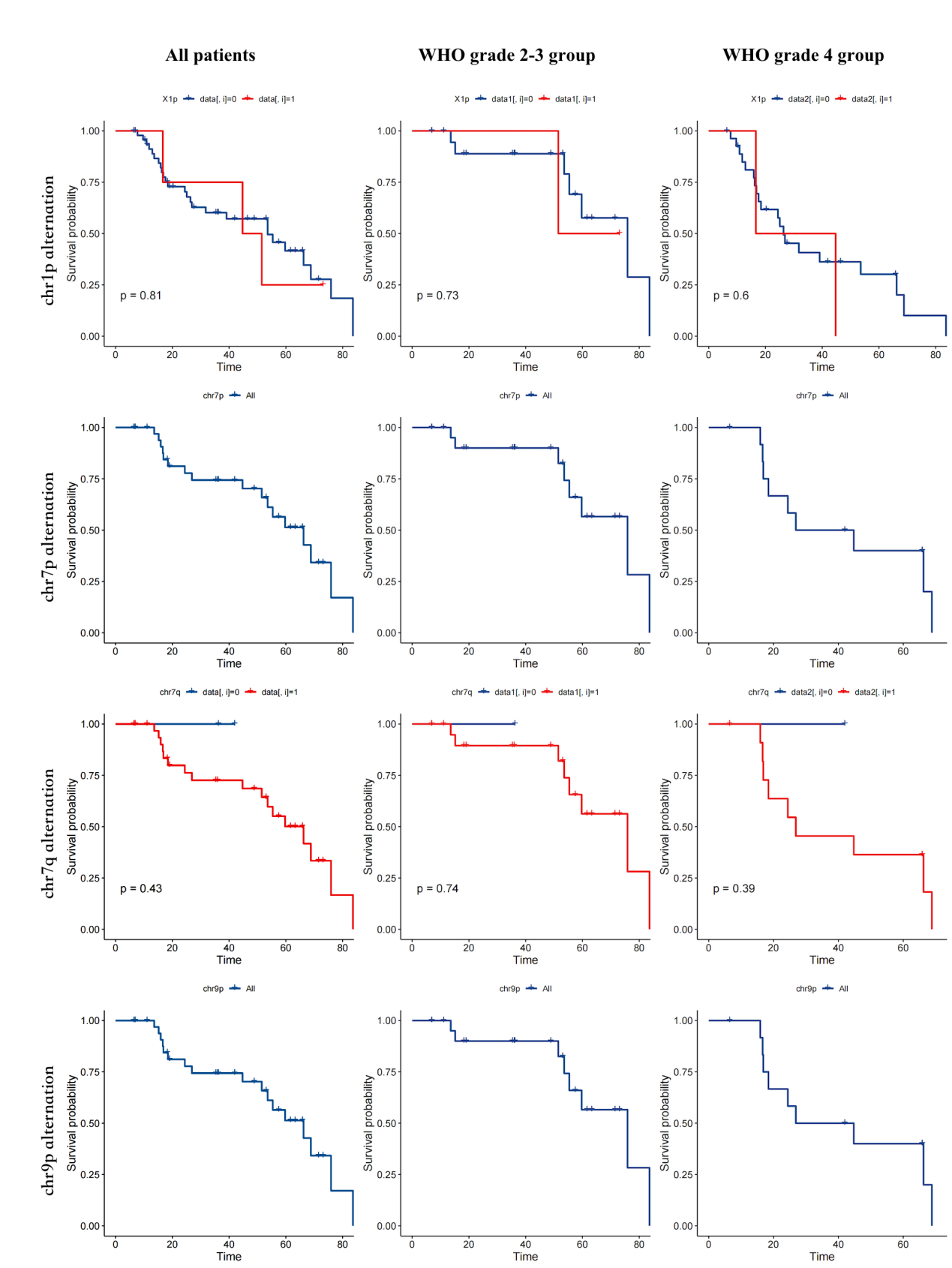

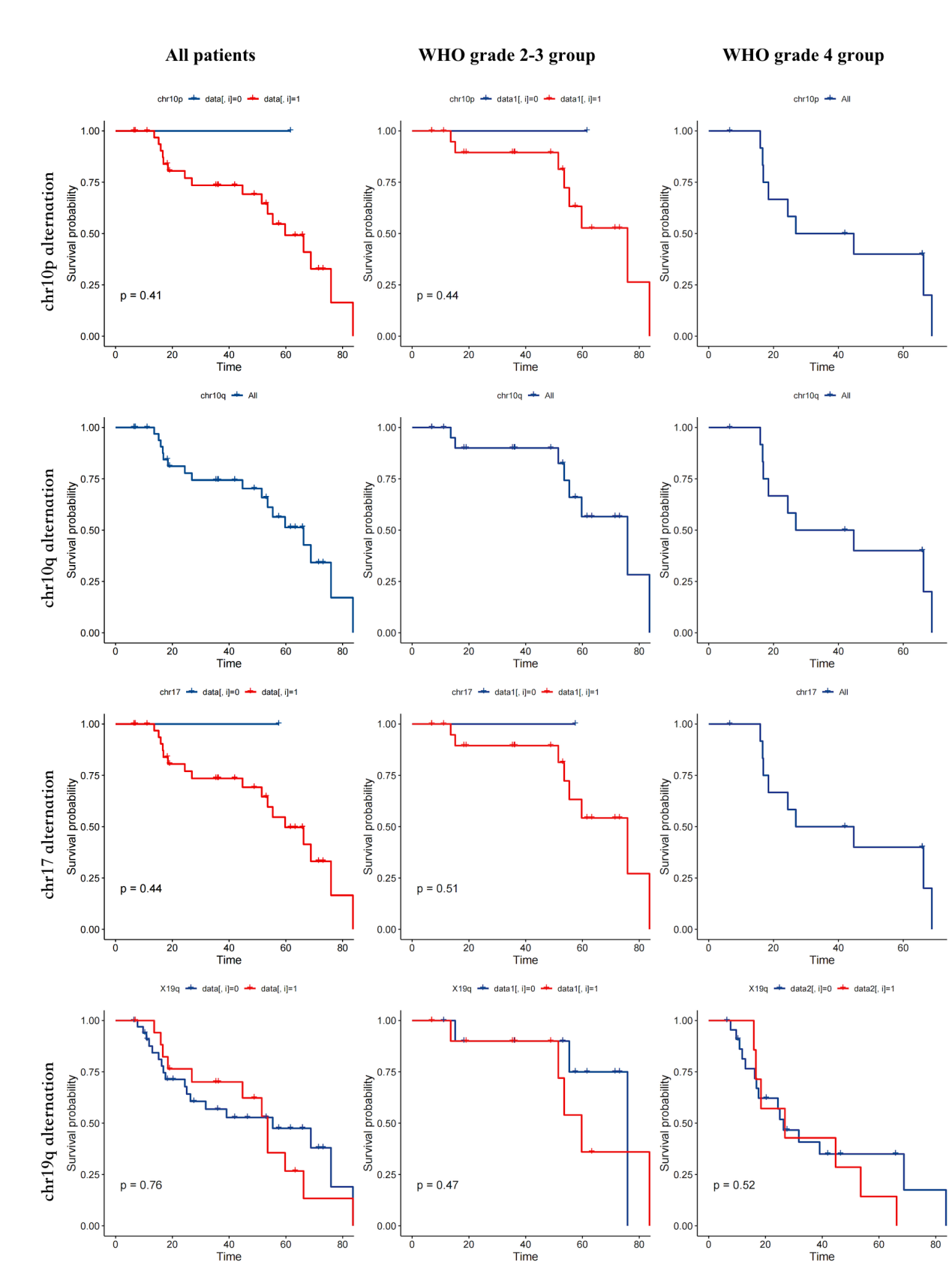


**detection**

Single-factor survival analysis was done for each factors included baseline characteristics and molecular alternations in this study. For continuous variables, the median value was set as cut-off value. The 3 columns was all patients, WHO grade 2-3 group and WHO grade 4 group respectively, and legends were shown on the top. Legend of each row was shown in the left. Lengeds for survival curves was listed below:

Age, blue line was age<40, red line was age≧40;

Sex, blue line was female, red line was male;

Body mass index (BMI), blue line was BMI<24, red line was BMI≧24;

Charlson comorbidity index (CCI), blue line was CCI>1, red line was CCI=0 or 1;

Recurrent tumor, blue line was newly diagnosis glioma, red line was recurrent glioma;

Karnofsky performance status (KPS), blue line was KPS<90, red line was KPS≧90;

Time between detection of tumor and surgery, blue line was the time between detection of tumor and surgery≧1 week, red line was the time between detection of tumor and surgery<1 week;

Tumor size, blue line was tumor diameter<5.5cm, red line was tumor diameter≧5.5cm;

Eloquent area, blue line was non-eloquent area tumor, red line was eloquent area tumor;

Necrosis area in MRI, blue line was no necrosis area shown in MRI, red line was necrosis area existed in MRI;

Peritumoral edema in MRI, blue line was no peritumoral edema area shown in MRI, red line was peritumoral edema existed in MRI;

Symptom of high intracranial pressure, blue line means patient without symptoms of high intracranial pressure, red line means patient had symptoms of high intracranial pressure;

Neurological symptoms, blue line means patient without neurological symptoms, red line means patient had neurological symptoms;

Seizure, blue line means patient did not have seizure before neurosurgery, red line means patient suffered from seizure before neurosurgery;

Gross total resection (GTR), blue line was subtotal resection or biopsy, red line was GTR of glioma;

Treated with stupp regime, blue line was not treated with standard Stupp regime, red line was treated with standard Stupp regime;

IHC of ATRX, immunohistochemistry examination of ATRX, blue line was negative, red line was positive;

IHC of CD68, immunohistochemistry examination of CD68, blue line was negative, red line was positive;

IHC of CD34, immunohistochemistry examination of CD34, blue line was negative, red line was positive;

IHC of EGFR, immunohistochemistry examination of EGFR, blue line was negative, red line was positive;

IHC of GFAP, immunohistochemistry examination of GFAP, blue line was negative, red line was positive;

IHC of H3K27M, immunohistochemistry examination of H3K27M, blue line was negative, red line was positive;

IHC of IDHI, immunohistochemistry examination of IDHI, blue line was negative, red line was positive;

IHC of NeuN, immunohistochemistry examination of NeuN, blue line was negative, red line was positive;

IHC of NF, immunohistochemistry examination of NF, blue line was negative, red line was positive;

IHC of Oligo_2, immunohistochemistry examination of Oligo_2, blue line was negative, red line was positive;

IHC of P53, immunohistochemistry examination of P53, blue line was negative, red line was positive;

IHC of S100, immunohistochemistry examination of S100, blue line was negative, red line was positive;

IHC of Syn, immunohistochemistry examination of Syn, blue line was negative, red line was positive;

IHC of Ki67, immunohistochemistry examination of Ki67 index, blue line was 0, red line was Ki67≧1%;

For gene alternation, blue line was wildtype, red line was alternation type. Survival was performed for gene listed in Supplementary table 1. Each gene was labeled in the left. “chr” was shorted for chromosome.

* None of tumors in WHO grade 2-3 group was stained by immunohistochemistry of NF
