# Supplementary material for: Clinical features, MRI, molecular alternations, and prognosis of astrocytoma based on WHO 2021 classification of central nervous system tumors: A single‐center retrospective study
Source: Cancer Med. 2024 Jul 5;13(13):e7369. doi: 10.1002/cam4.7369 (PMC11226410; doi:10.1002/cam4.7369)
Supplement: Supplementary file 3 — Table S1. [file CAM4-13-e7369-s004.docx]

Supplementary table 1 Molecular list for integrate diagnosis

| ACVR1  ATRX  BCOR  BRAF  CDK4  CDK6  CDKN2A  CDKN2B  CIC  EGFR  FBXW7  FGFR1  FGFR2  FGFR3  FGFR4  FUBP1  H3F3A  HIST1H3B  HIST1H3C  IDH1  IDH2  KIT  KMT5B  KRAS  MAP2K1  MET  MYB  MYBL1  MYC  MYCN  NF1  NOTCH1  NRAS  NTRK2  NTRK3  PDGFRA  PEG3  PIK3CA  PIK3CB  PIK3R1  PPM1D  PTEN  PTPN11  RB1  SMARCA4  SMARCB1  TERT  TOP3A  TP53  TSC1  TSC2  YAP1  chr1p  chr7p  chr7q  chr9p  chr10p  chr10q  chr17  chr19q |
| --- |
