# Supplementary material for: Clinical features, MRI, molecular alternations, and prognosis of astrocytoma based on WHO 2021 classification of central nervous system tumors: A single‐center retrospective study
Source: Cancer Med. 2024 Jul 5;13(13):e7369. doi: 10.1002/cam4.7369 (PMC11226410; doi:10.1002/cam4.7369)
Supplement: Supplementary file 4 — Table S2. [file CAM4-13-e7369-s003.docx]

Supplementary table 2

|  | WHO 2-3 | WHO 4 | *P* value |
| --- | --- | --- | --- |
| Ki_67 | 7.27±1.53 | 36.65±5.23 | P＜0.001* |
| IDH1_IHC | 13/15 | 12/17 | 0.412 |
| ATRX_IHC | 8/14 | 7/11 | 0.697 |
| P53_IHC | 19/24 | 19/22 | 0.457 |
| GFAP_IHC | 25/25 | 25/26 | >0.999 |
| Oligo_2_IHC | 15/15 | 15/16 | 0.488 |
| H3K27M_IHC | 0/5 | 0/3 | - |
| NeuN_IHC | 7/16 | 6/17 | 0.638 |
| S_100_IHC | 21/22 | 18/19 | >0.999 |
| Syn_IHC | 12/12 | 11/12 | >0.999 |
| NF_IHC | 0/0 | 1/4 | >0.999 |
| CD34_IHC | 19/23 | 17/18 | 0.606 |
| CD68_IHC | 2/2 | 3/3 | - |
| EGFR_IHC | 15/17 | 13/13 | >0.999 |
| ACVR1 | 0/27 | 1/15 | 0.378 |
| ATRX | 19/27 | 10/15 | 0.716 |
| BCOR | 0/27 | 1/15 | 0.361 |
| BRAF | 15/27 | 10/23 | 0.741 |
| CDK4 | 18/27 | 9/15 | >0.999 |
| CDK6 | 11/27 | 12/15 | 0.004* |
| CDKN2A | 6/27 | 15/15 | P＜0.001* |
| CDKN2B | 10/27 | 15/15 | P＜0.001* |
| CIC | 4/27 | 1/15 | >0.999 |
| EGFR | 9/27 | 8/15 | 0.763 |
| FBXW7 | 0/27 | 1/15 | 0.361 |
| FGFR1 | 10/27 | 7/15 | 0.265 |
| FGFR2 | 6/27 | 9/15 | 0.003* |
| FGFR3 | 8/27 | 4/15 | >0.999 |
| FGFR4 | 9/27 | 6/15 | 0.474 |
| FUBP1 | 1/27 | 0/15 | >0.999 |
| H3F3A | 0/27 | 0/15 | - |
| HIST1H3B | 0/27 | 1/15 | 0.361 |
| HIST1H3C | 0/27 | 0/15 | - |
| IDH1 | 27/27 | 31/33 | 0.497 |
| IDH2 | 1/21 | 2/25 | >0.999 |
| KIT | 10/27 | 5/15 | >0.999 |
| KMT5B | 6/27 | 0/15 | 0.274 |
| KRAS | 17/27 | 8/15 | 0.681 |
| MAP2K1 | 0/27 | 0/15 | - |
| MET | 8/27 | 4/15 | >0.999 |
| MYB | 9/27 | 6/15 | 0.150 |
| MYBL1 | 7/27 | 5/15 | 0.693 |
| MYC | 10/27 | 9/15 | 0.004* |
| MYCN | 2/27 | 4/15 | 0.328 |
| NF1 | 0/27 | 0/15 | - |
| NOTCH1 | 13/27 | 6/15 | 0.587 |
| NRAS | 0/27 | 1/15 | 0.361 |
| NTRK2 | 17/27 | 6/15 | 0.265 |
| NTRK3 | 7/27 | 5/15 | 0.127 |
| PDGFRA | 12/27 | 7/15 | 0.137 |
| PEG3 | 10/27 | 6/15 | 0.393 |
| PIK3CA | 19/27 | 13/15 | 0.708 |
| PIK3CB | 1/27 | 0/15 | >0.999 |
| PIK3R1 | 2/27 | 2/15 | 0.609 |
| PPM1D | 5/27 | 5/15 | 0.422 |
| PTEN | 16/27 | 11/15 | 0.452 |
| PTPN11 | 13/27 | 3/15 | 0.153 |
| RB1 | 8/27 | 7/15 | 0.166 |
| SMARCA4 | 3/27 | 1/15 | 0.288 |
| SMARCB1 | 0/27 | 0/15 | - |
| TERT | 3/27 | 4/32 | >0.999 |
| TOP3A | 11/27 | 4/15 | 0.319 |
| TP53 | 25/27 | 12/15 | 0.539 |
| TSC1 | 0/27 | 0/15 | - |
| TSC2 | 3/27 | 0/15 | 0.525 |
| YAP1 | 1/27 | 0/15 | >0.999 |
| 1p | 2/27 | 3/32 | >0.999 |
| 19q | 15/27 | 7/32 | 0.030* |
| chr7p | 6/27 | 15/15 | - |
| chr7q | 26/27 | 14/14 | >0.999 |
| chr9p | 4/27 | 15/15 | - |
| chr10p | 27/27 | 15/15 | >0.999 |
| chr10q | 5/27 | 15/15 | - |
| chr17 | 26/27 | 15/15 | >0.999 |
